# Early loss of *Scribble* affects cortical development, interhemispheric connectivity and psychomotor activity

**DOI:** 10.1038/s41598-021-88147-1

**Published:** 2021-04-27

**Authors:** Jerome Ezan, Maité M. Moreau, Tamrat M. Mamo, Miki Shimbo, Maureen Decroo, Melanie Richter, Ronan Peyroutou, Rivka Rachel, Fadel Tissir, Froylan Calderon de Anda, Nathalie Sans, Mireille Montcouquiol

**Affiliations:** 1 Université de Bordeaux, INSERM, Neurocentre Magendie, U1215, 33077 Bordeaux, France; 2 Germany Center for Molecular Neurobiology Hamburg (ZMNH), Research Group Neuronal Development, University Medical Center Hamburg-Eppendorf, Hamburg, Germany; 3 Neurobiology-Neurodegeneration and Repair Laboratory, National Eye Institute, NIH, Bethesda, MD 20892 USA; 4 Developmental Neurobiology Group, Institute of Neuroscience, University of Louvain, Avenue Mounier 73, Box B1.73.16, 1200 Brussels, Belgium

**Keywords:** Neuroscience, Development of the nervous system, Diseases of the nervous system, Genetics of the nervous system, Learning and memory

## Abstract

Neurodevelopmental disorders arise from combined defects in processes including cell proliferation, differentiation, migration and commissure formation. The evolutionarily conserved tumor-suppressor protein Scribble (Scrib) serves as a nexus to transduce signals for the establishment of apicobasal and planar cell polarity during these processes. Human *SCRIB* gene mutations are associated with neural tube defects and this gene is located in the minimal critical region deleted in the rare Verheij syndrome. In this study, we generated brain-specific conditional cKO mouse mutants and assessed the impact of the *Scrib* deletion on brain morphogenesis and behavior. We showed that embryonic deletion of *Scrib* in the telencephalon leads to cortical thickness reduction (microcephaly) and partial corpus callosum and hippocampal commissure agenesis. We correlated these phenotypes with a disruption in various developmental mechanisms of corticogenesis including neurogenesis, neuronal migration and axonal connectivity. Finally, we show that *Scrib* cKO mice have psychomotor deficits such as locomotor activity impairment and memory alterations. Altogether, our results show that *Scrib* is essential for early brain development due to its role in several developmental cellular mechanisms that could underlie some of the deficits observed in complex neurodevelopmental pathologies.

## Introduction

The mammalian brain, seat of cognitive and behavioral processing, is the result of numerous, complex but coordinated mechanisms of development. Patients with disruptions in fundamental processes, such as proliferation, migration, polarity, branching or synaptogenesis will typically exhibit neurodevelopmental disorders. As a result, primary microcephaly^1^, improper cortical layering^2^ or commissural defects such as agenesis of the corpus callosum (ACC)^3^ are frequently associated with neurobehavioral disorders including Intellectual Disabilities, Autism Spectrum Disorders (ASDs), epilepsy and/or Attention Deficit Hyperactivity Disorders (ADHD). Rare Copy-Number Variants (CNVs) associated with these disorders are found in specific regions of the human genome (including 8q24.3) and may impact these processes^4^. In order to understand the basis of such neurodevelopmental and neuropsychiatric disorders, it is essential to decipher the genetic, molecular and cellular mechanisms that govern brain development^5^.

Scribble (Scrib) is a conserved scaffold protein that acts as a hub for several signaling pathways and that functions in cell polarity during development^6,7^. In rodents, a homozygous *Scrib* mutation in a spontaneous mutant called *Circletail* (*Crc*) leads to early lethality accompanied by a severe form of neural tube defects (NTDs)^8^. This mutant displays tissue polarity defects^9^ that are hallmarks of planar cell polarity (PCP) signaling pathway deregulation^10^, which can ultimately impact on the development and function of the nervous system^11–13^. Beyond its role in epithelial cells, *Scrib* has been shown to participate in motor neuron migration^14^ and axonal guidance in the hindbrain in zebrafish^15^ but also central nervous system myelination in rodents^16^. Additionally, our group showed a role for *Scrib* in the brains of adult mice in fine tuning of excitatory synapses and correlated its deletion with some features of ASDs^17–19^.

In humans, the absence or mutation of the *SCRIB* gene is linked to neurodevelopmental disease. Mutation in SCRIB is associated with NTDs (OMIM # 182940)^20–23^, like other core PCP genes^24,25^. NTDs represent a CNS (Central Nervous System) congenital malformations that affect 1:1000 children^26^*.* Spina bifida (spinal NTD) remains the commonest congenital CNS defect and is often associated with cerebellum, corpus callosum abnormalities and hydrocephalus^27^. As a result, patients with spina bifida face neurobehavioural alterations that include psychosocial, memory and motor defects^28^. In addition, microdeletions in 8q24.3 (encompassing both *PUF60* and *SCRIB*) were found in children presenting microcephaly^29^. 8q24.3 deletion syndrome, also called Verheij syndrome (VRJS, OMIM #615583), is characterized by complex features such as growth retardation, short stature, dysmorphic facial features as well as renal and cardiac defects^29,30^. The neurological symptoms of this syndrome include delayed psychomotor development, mild intellectual disability and epilepsy that are frequently associated with neurodevelopmental defects such as microcephaly and/or ACC^29,30^. Patients with a large deletion on chromosome 8 show microcephaly and ACC^31^, and a more specific deletion of the 8q24.3 region is associated with ASDs or ADHD^32^. VRJS is considered as a contiguous gene syndrome because it results from the haploinsufficiency of the *SCRIB* and *PUF60* genes, which are located within the minimal critical region^29^. The best support for *Scrib* participating in VRJS came from morpholino-based knockdown experiments in zebrafish showing altered brain size in either *scrib* or *puf60* morphants^29^. While all these data suggest a role for *Scrib* in early vertebrate brain development, the potential contribution of *Scrib* to some of the observed structural and psychomotor deficits has never been assessed in mammals.

To evaluate the structural and psychomotor consequences of early *Scrib* deletion in the mammalian brain, we developed a conditional gene-targeting strategy to generate two mouse lines. The results show that early deletion of *Scrib* in the dorsal telencephalon lead to (1) reduced brain cortical size associated with cortical layering defects (2) agenesis of the corpus callosum (CC) and the hippocampal commissure, stemmed from a combination of neurogenesis and neuronal migration defects. Behavioral analysis of *Scrib* cKO animals show altered psychomotor behavior accompanied by memory deficits. Altogether, our results show that the absence of *Scrib* during early brain development lead to differential structural and behavioral deficits, often observed in spina bifida or VRJS patients, supporting a role for this gene in these pathologies.

## Results

### *Scrib* expression is consistent with a developmental function in the forebrain

**Figure 1:**
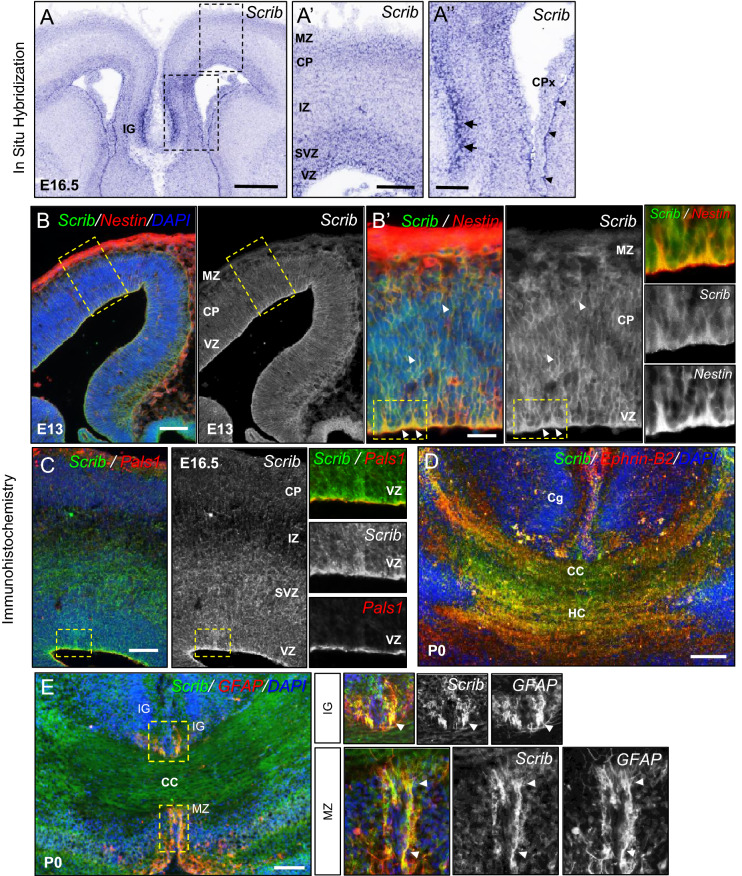
Scrib expression in the mouse developing forebrain. (**A-A″**) Representative *Scrib* mRNA expression pattern by ISH on E16.5 mouse embryo sections. The dashed boxes in A are magnified in (**A′**) and (**A″**). Scrib mRNA is detected on the cortical plate (CP), in the subventricular zone (SVZ), the ventricular zone (VZ) and in the indusium griseum (IG, arrows) and in ependymal cells (arrowheads) next to the choroid plexus (CPx). *MZ* marginal zone, *IZ* intermediate zone. Scale bar 0.5 mm (**A**) and 0.1 mm (**A**′–**A″**). (**B**) Representative Scrib (green) and Nestin (a general marker of neural progenitor cells, red) expression pattern by IF on E13 mouse embryo coronal sections. The dashed boxes in (**B**) are magnified in (**B**′), then within the insets. Scrib protein is detected throughout the cortical plate (CP) and is enriched in the VZ. Scale bar 0.1 mm (**B**), 0.05 mm (**B′**). (**C**) Representative Scrib (green) and Pals1 (an apical surface marker, red) expression pattern by IF on E16.5 mouse embryo coronal sections. The higher magnification illustrates Scrib accumulation at cell–cell contacts in the entire VZ, next to the apical marker Pals-1 (red). Scale bar 0.1 mm. (**D**,**E**) Representative Scrib (green) expression pattern by IF on P0 cortex (**C**), Corpus Callosum (CC) (**D**) and midline glia (**E**). Scrib labeling overlaps with Ephrin-B2 (marker of the CC, red in (**D**) but is also markedly enriched in GFAP-positive (red in **E**) structures including the indusium griseum (IG) and the midline zipper (MZ). Cg is the cingulate cortex. Higher magnification for selected insets (boxed areas) illustrates strong Scrib expression in glial midline structures (see arrowheads). Scale bar 0.1 mm (**D**,**E**).

We evaluated the expression profile of Scrib in the developing brain using in situ hybridization (ISH) (Fig. 1A) and an in-house Scrib antibody for immunohistochemistry (Fig. 1B–E). At E16.5, Scrib ISH indicated that it was expressed throughout the dorsal forebrain and enriched in specific regions such as the upper layers of the cortical plate (CP) along the medio-lateral axis (from the cingulate to the piriform cortex), in the sub-ventricular and ventricular zones (SVZ and VZ, respectively) and the Indusium Griseum (IG) at the cortical midline (Fig. 1A-A″). Right before the neurogenesis peak, at E13, Scrib in expressed in most neural Nestin-positive progenitors throughout the cortical plate and is enriched in the VZ (Fig. 1B). Scrib immunostaining at E16.5 is consistent with its mRNA expression pattern, with an enrichment in the CP and VZ at cell–cell junctions in both apical (labeled by Pals1) and basal domains (Fig. 1C). At birth, Scrib is also found in axonal tracts, especially in the interhemispheric commissural fibers (Fig. 1D). Double staining of Scrib (green) and GFAP (glial fibrillary acidic protein, a marker of mature glial cells, red) revealed that Scrib is also enriched in midline glia cells (Fig. 1E).

### Early *Scrib* deletion results in microcephaly

**Figure 2:**
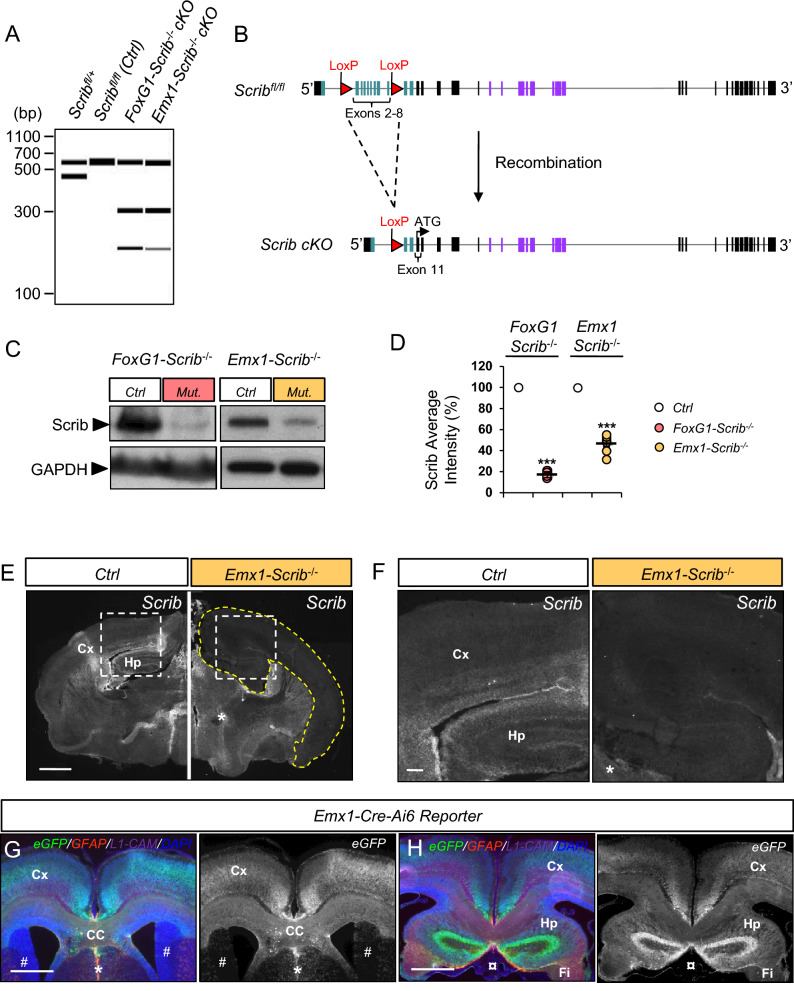
Generation and characterization of *Scrib* conditional knockout mouse mutants. (**A**) Virtual gel of PCR genotyping to detect wild-type (437 bp), floxed (541 bp) and targeted cKO (193 bp) alleles. *Cre*-mediated excision was confirmed in P0 cortices by the presence of a 300 bp product (lanes 3 and 4). In absence of Cre, the wild-type and floxed alleles remain intact as determined by 437 and 541 bp fragments, respectively (lane 1 and 2). When Cre-mediated recombination occurs (lanes 3 and 4), the floxed allele is excised, resulting in a 193 bp band, suggesting efficient recombination. (**B**) Schematic representation of the genomic organization of mouse *Scrib* gene with 38 exons (solid boxes) including exons encoding for LRR (green boxes) or PDZ (purple boxes) domains of the protein. (**C**) Representative western blots from control and *Scrib*^−/−^ cKO cerebral cortices showing full-length Scrib protein. Cortical protein extracts of P0 *FoxG1-Scrib*^−/−^ and *Emx1-Scrib*^−/−^ cKOs were immunoblotted with anti-Scrib antibody and anti-GAPDH as a control. cKO lysates (lanes 2 and 4) show reduced levels of Scrib when compared with control (lanes 1 and 3). (**D**) Scatterplots summarizing western blot quantification (densitometric intensity values normalized to the control). Statistical analysis via a two-tailed t test (P** < 0.001, P*** < 0.0001) using between 4 and 8 cortical samples per genotype from at least 3 independent experiments. Error bars indicate the SEM. (**E**) Scrib expression on coronal cryosection of P0 *Emx1-Scrib*^−/−^ cKOs mutants and control littermates. A dramatic decrease in Scrib expression is observed specifically in cortex and hippocampus (area surrounded by yellow dashed line). Persistence of Scrib expression in the ventro-medial structures verifies the specificity of the excision (see star*). Scale bar 0.5 mm. (**F**) Higher magnification insets for the Hp and Cx areas from (**E**). Scale bar 0.1 mm. (**G**,**H**) P0 coronal sections of *Emx1-Cre-Ai6* mouse brains. By crossing *Emx1-Cre* with an Ai6 reporter mouse strain, we confirmed the *Emx1*-driven expression of the Cre recombinase in the cortex (Cx), the corpus callosum (CC), the hippocampus (Hp) and the fimbria (Fi). Both rostral (**G**) and caudal (**H**) sections show robust ZSGreen1 (eGFP) expression in the dorsal forebrain assessing efficient recombination, including in GFAP-positive cells from the midline glia but also in the L1-CAM axonal tracts of the CC. On the other hand, the most ventral regions including the striatum (see star *), the mediolateral neuroepithelium (see #) or the choroid plexus (see ¤) show almost no sign of recombination. Scale bar 0.5 mm.

**Figure 3:**
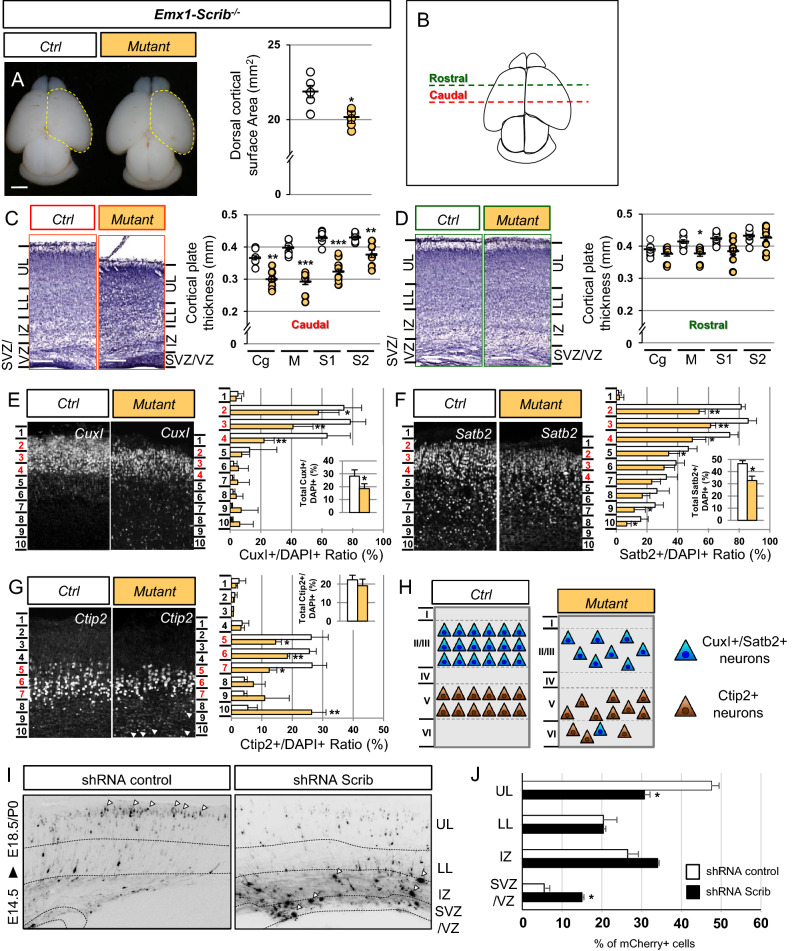
Early deletion of *Scrib* leads to microcephaly associated with cortical layering and neuronal migration defects. (**A**) Dorsal views of P0 *Emx1-Scrib*^−/−^ cKO brains. Dorsal cortical surface areas are outlined with a yellow dashed line. Statistical analysis via a two-tailed t test (*P < 0.05) using between 5 and 7 brains per genotype from at least 3 independent experiments. Error bars indicate the SD. Scale bar 1 mm. (**B**) Schematic view of a P0 brain sectioned coronally at the rostral (green dotted line) or caudal (red dotted line) level. (**C**,**D**) Representative hematoxylin staining of coronal sections from newborn *Emx1-Scrib*^−/−^ cKO motor cortex at the caudal (**C**, red labels) and rostral (**D**, green labels) levels and their respective controls. A marked reduction of the caudal motor cortex thickness (M) in cKOs extends to the cingulate (Cg) and somatosensory (S1, S2) cortex. No major difference was observed at the rostral level. Cortical plate thickness was measured radially from the top of the upper layer (UL) to the bottom of the lower layer (LL) of the cortex. *IZ* intermediate zone, *SVZ* sub-ventricular zone, *VZ* ventricular zone. Statistical analysis via a two-tailed t test (**P < 0.01, ***P < 0.001) using 8 measurements per genotype from at least 3 independent experiments. Error bars indicate the SEM. Scale bar 0.2 mm. (**E–G**) Representative immunofluorescence staining of CuxI (**E**), Satb2 (**F**) and Ctip2 (**G**) on coronal sections from newborn *Emx1-Scrib*^−/−^ cKO brains in the caudal motor cortex. Quantification of CuxI-, Satb2- and Ctip2-positive neurons is shown as a percentage (see “Materials and methods”). The proportion of CuxI- and Satb2-positive neurons is decreased in *Emx1-Scrib*^−/−^ cKO brains, while the Ctip2 percentage is unchanged (see insets). Several ectopic Ctip2-positive cells are mislocalized in lower bins (white arrowheads). Statistical analysis via a two-tailed t test (*P < 0.05, **P < 0.01) using between 3 and 4 measurements per genotype from at least 3 independent experiments. Error bars indicate the SD. (**H**) Schematic representation of cortical layering in the caudal motor cortex of *Emx1-Scrib*^−/−^ cKO and its control. Early-born neurons (brown) are mislocalized, while late-born neurons (blue) are decreased in proportion suggesting both neurogenesis and migration defects after early loss of Scrib function. See also Supplementary Fig S2. (**I**) Cortical neurons were electroporated in utero at E14.5 with an mCherry expressing vector together with control shRNA or a previously validated Scrib shRNA. Brains were fixed at E18.5/P0. Nuclei were stained with DAPI (not shown) in order to delineate cortical subregions: dotted lines represent boundaries between the upper layers (UL) and lower layers (LL) of the cortex, the intermediary zone (IZ), the subventricular zone (SVZ) and the ventricular zone (VZ). Arrowheads indicate either neurons reaching the upper layers of the cortex in the control condition or Scrib shRNA-electroporated cells that remain in the deepest layers of the cortex. (**J**) Quantification of the distribution of mCherry-positive cells in distinct subregions of the cerebral cortex for each condition (shRNA control, white bar; Scrib shRNA, black bar). Analysis was performed using at least 3 independent experiments. Error bars indicate SD. Statistical analysis via a two-tailed *t* test (*P < 0.01) per condition from at least 3 independent experiments.

Scrib spontaneous mouse mutant *Circletail* causes severe brain and neural tube damages that result in neonatal lethality^8^, precluding the analysis of the role of *Scrib* during forebrain development^17^ (Fig S1). In order to circumvent this issue, we have applied a conditional gene-targeting strategy to inactivate *Scrib* at different developmental stages and in different cellular types in the brain. We crossed floxed *Scrib*^*fl/fl*^ mice with either *Emx1*-Cre mice, which express the Cre recombinase starting at E10.5 in the dorsal telencephalic progenitors, or *FoxG1*-Cre mice, which express the Cre recombinase as early as E8.5 in the entire telencephalon (hereafter reported as *Emx1-Scrib*^−/−^ and *FoxG1-Scrib*^−/−^ cKOs respectively). Specific *Scrib* excision in conditional mutants was validated (Fig. 2A–F), and the spatial expression pattern of Cre recombinase was further confirmed by crossing *Emx1-Cre* mice with the Ai6 reporter mice (Fig. 2G–H). The cerebral hemispheres of *Emx1-Scrib*^−/−^ cKOs were 7.8% ± 0.5% smaller than those of their control littermates, a mild but significant reduction (Fig. 3A). Histological brain analysis at the rostral and caudal levels, revealed a marked decrease in the thickness of the caudal cerebral cortex (~ 25% throughout the mediolateral axis) (Fig. 3B). This decrease was observed throughout the cingulate (Cg), motor (M), primary somatosensory (S1) and secondary somatosensory (S2) cortices and was maintained in adults (data not shown). This phenotype was absent in more rostral regions of *Emx1-Scrib*^−/−^ cKO brains (Fig. 3C). *FoxG1-Scrib*^−/−^ mutant brains displayed a similar but more severe reduction of cortical area and thickness along the entire rostrocaudal axis (Fig S2A–D). We next sought to identify the cellular roots of the microcephaly deficits observed in *Scrib* mutant mice.

### Loss of *Scrib* disrupts cortical layering

To evaluate the consequences of *Scrib* deletion on cortical layering, we examined the expression of neuronal markers such as CuxI (layer II/III), Satb2 (layer II/VI) and Ctip2 (layer V), some of these markers being essential to the formation of the CC^33^. In *Emx1-Scrib*^−/−^ mutant P0 pups, we observed a reduction in the numbers of CuxI-positive cells (ctrl, 28.2% ± 4.8; mutant, 18.5% ± 3.7; *p* = 0.04) and Satb2-positive cells (ctrl, 46.6% ± 2.4; mutant, 32.7% ± 3.5; *p* = 0.002) (Fig. 3E,F). This was particularly pronounced in bins 2–4, corresponding to layers II–III. In contrast, the expression of Ctip2, a marker of early-born neurons, was unchanged (ctrl, 22.3% ± 2.5; mutant, 19.1% ± 1.8; *p* = 0.24), but its cortical distribution was altered: many Ctip2-positive neurons were mislocalized in the deeper layers of the motor cortex (bins 8–10), implying migration defects (Fig. 3G,H). Similar results were observed within the somatosensory cortex (data not shown). A comparative analysis in *FoxG1-Scrib*^−/−^ mutant mice showed a more severe phenotype than that of *Emx1-Scrib*^−/−^ mutants, with a dramatic reduction in CuxI, Satb2 and Ctip2 expression (Fig S2E–H). Using in utero electroporation approach employing a previously validated shRNA construct^17^, we depleted *Scrib* in E14.5 brain embryos, and observed a significant increase in the fraction of electroporated cells mislocalized in the VZ/SVZ (ctrl shRNA, 5.4% ± 0.5; Scrib shRNA, 15% ± 1.4) associated with a significant decrease in the fraction of cells reaching the upper layers (UL) (ctrl shRNA, 47.1% ± 1.4; Scrib shRNA, 30.7% ± 1.9) (Fig. 3I,J).

Altogether, these results indicate that embryonic deletion of *Scrib* alters the neuronal composition of the cortical layers of the brain of the mutant mice, a phenotype that could stem from a decrease in neurogenesis and/or a reduction in neuronal migration.

### Proliferation defects in cortical progenitors in *Emx1-Scrib*^*−/−*^ mutant

**Figure 4:**
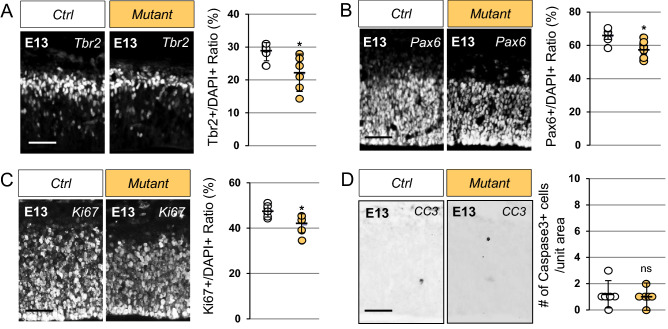
Reduction in the number of neural precursor cells in *Emx1-Scrib*^−/−^ mutant cortices is associated with proliferation but not apoptosis defects. (**A**–**D**) Representative immunostaining of Tbr2 (**A**), Pax6 (**B**), Ki67 (**C**) and activated cleaved caspase 3 (CC3) (**D**) on coronal sections from embryonic stage E13 *Emx1-Scrib*^−/−^ cKO brains. Cells were counted in 312.35 μm-wide cortical areas from three mutants and three controls from two different litters. Quantification of Tbr2-, Pax6- and Ki67-positive cells is shown as a percentage (see “Materials and methods”). The proportion of Tbr2- and Pax6-positive progenitors as well as Ki67-positive cells is decreased in *Emx1-Scrib*^−/−^ cKO embryonic brains. No variation of activated caspase3-positive apoptotic cells is observed in *Emx1-Scrib*^−/−^ mutant cortices. Statistical analysis via a two-tailed t test (*P < 0.05) using 6 measurements per genotype. Error bars indicate the SD. Scale bar 50 μm.

To assess potential progenitor proliferation deficits, we examined the numbers of both apical (using Pax6 as a marker)^34^ and basal progenitors (using Tbr2 as a marker)^35^ in cortical sections of E13 control and mutant littermates. We found a significant decrease in the numbers of Tbr2-positive neuronal progenitor cells in the VZ (ctrl, 28.6% ± 2.4; mutant, 22.0% ± 5.3; *p* = 0.027) and Pax6-positive intermediate basal progenitors in the SVZ (ctrl, 65.8% ± 4.5; mutant, 57.5% ± 5.3; *p* = 0.015) in the *Emx1-Scrib*^−/−^ mutant neocortex (Fig. 4A,B). We next used an antibody against Ki67 to assess the levels of proliferation in absence of *Scrib*. As shown in Fig. 4C, the number of Ki67-positive cells was also slightly reduced in cKO neocortices (ctrl, 47.7% ± 2.7; mutant, 41.9% ± 4.3; *p* = 0.021), suggesting a mild proliferation decrease of progenitors. Finally, we evaluated apoptosis and did not detect significant variation of cell death in in *Emx1-Scrib*^−/−^ mutant cortices (Fig. 4D). Together, these results demonstrate that the loss of *Scrib* lead to a mild decrease in progenitors proliferation, leading to a small reduction of neuronal progenitor populations in the VZ/SVZ, but has no impact on apoptosis levels.

### Early deletion of *Scrib* affects corpus callosum and hippocampal commissure development

**Figure 5:**
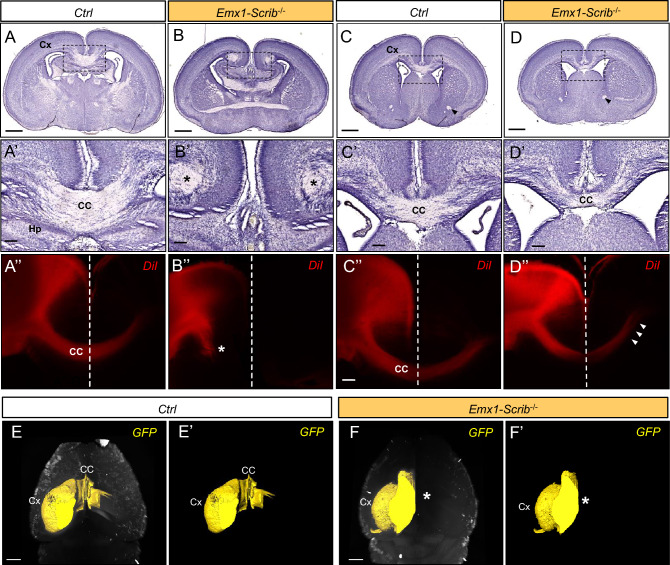
Partial corpus callosum agenesis in *Emx1-Scrib*^−/−^ *cKO* mutants. (**A**–**D**) Representative hematoxylin staining of coronal sections from newborn *Emx1-Scrib*^−/−^ cKO brains (**B**,**D**) and their respective controls (**A**,**C**) at the caudal (**A**,**B**) or rostral (**C**,**D**) levels. Dashed boxes in (**A**,**D**) are magnified in (**A′**–**D′**). (**A′–D′**) Higher magnification for selected insets (boxed areas) from (**A**–**D**) illustrating high penetrance of CC agenesis (ACC) at the caudal level. At P0, 93% of *Emx1-Scrib*^−/−^ (n = 28) cKO brains displayed ACC. Instead of crossing the midline, CC axons formed whorls (Probst bundles, PB) on either side of the midline that are indicated with an asterisk. Compared with control brains, a gap between hemispheres indicates fusion defects. Despite an apparent thinning, CC fibers do cross the midline at the rostral level. (**A″–D″**) DiI crystals placed in the dorsomedial cortex trace CC axons in *Emx1-Scrib*^−/−^ cKO brains (**B″**,**D″**) and their respective controls (**A″**,**C″**) at the caudal (**A″**–**B″**) or rostral (**C″**–**D″**) levels at P0. *Emx1-Scrib*^−/−^ cKO brains displayed occasional stalled fibers in some mutant brains (asterisk in **B″**). *Cx* cortex, *Hp* hippocampus, *CC* corpus callosum. The midline is indicated as a white dashed line. (**E–F′**) 3D imaging of adult brains cleared with uDISCO from *Emx1-Scrib*^−/−^ cKO brains (**F**,**F′**, orange) and their respective controls (**E**,**E′**). 3D reconstruction of the GFP expressed after viral infection in the sensory-motor cortex are represented in yellow. In *Emx1-Scrib*^−/−^ cKO brains, agenesis of the corpus callosum is confirmed by the absence of cortical fibers passing through the contralateral side (asterisk in **F′**). See also Supplementary Fig S3.

**Figure 6:**
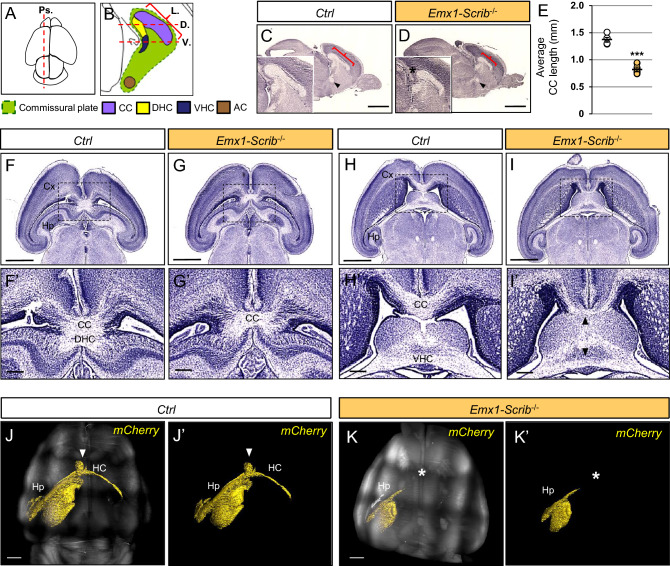
ACC is accompanied by hippocampal commissure agenesis in *Scrib*^−/−^ cKO mutants. (**A**,**B**) Schematic dorsal (**A**) or para-sagittal (**B**) views of a P0 mouse brain. Brains were sectioned either para-sagitally (Ps. in **A**) or horizontally (in **B**) as indicated by the red dashed line at the level of the Dorsal (DHC) or Ventral (VHC) Hippocampal Commissures. CC length (L.) was determined as indicated by the red bracket. (**C**,**D**) Marked reduction of CC length (see red brackets and asterisk) along the rostrocaudal axis in the brains of *Emx1-Scrib*^−/−^ (**D**, orange, n = 5) cKO brains as compared with those of their control littermates (**C**, white, n = 5). The Anterior Commisure (AC) is present in the mutant (black arrowhead). (**E**) Quantification of average CC length (in mm). Statistical analysis via a two-tailed *t* test (***P < 0.0001) using between 3 and 5 brains per genotype from at least 3 independent experiments. (**F–I**) Representative hematoxylin staining of serial horizontal sections from newborn *Emx1-Scrib*^−/−^ (**G**,**I**) cKO brains and their respective controls (**F**,**H**) at the dorsal (**F**–**G**) and ventral level (**H**–**I**) as defined in (**B**). (**F′–I′**) Higher magnification for selected insets (boxed areas) from (**F**–**I**) illustrating agenesis of the DHC (**G**′) and CC hypoplasia (**G′**,**I′**). Either dorsal (**F′**–**G′**) or ventral (**H′**–**I**′) sections showed showed some callosal and hippocampal axon bundles still crossing through the midline. (**J**,**K**) Light-sheet microscopy imaging of *Emx1-Scrib*^−/−^ cKO adult brains (**K**) and its control (**J**) cleared with uDISCO. 3D reconstruction of the mCherry expressed after viral infection in the CA3 region of the hippocampus cortex is represented in yellow. The HC (indicated by an arrowhead in control) is absent in brains from cKO mutants (asterisk in **K**). Scale bars 1 mm in (**C**,**D**′,**F**–**K**), 0.2 mm in (**F′**–**I′**). See also Supplementary Fig S5.

Histological analysis was performed along the rostrocaudal axis in order to assess the impact of early Scrib loss on the major forebrain commissure (the CC) but also the dorsal and ventral hippocampal commissure (DHC and VHC) and the anterior commissure (AC) (Fig. 5). Coronal histological sections of P0 *Emx1-Scrib*^−/−^ mutant brains revealed ACC in caudal sections, with callosal axons apparently unable to cross the midline (Fig. 5A,B). Failure of the axons to cross was accompanied by bundles of aberrantly projecting axons near the midline, known as Probst bundles, which are frequently associated with hemispheric fusion defects (Fig. 5A′-B′). Anterograde axonal tracing studies using DiI staining confirmed the absence of midline-crossing callosal axons (Fig. 5A″-B″). In contrast, callosal fibers in rostral sections from the brains of *Emx1-Scrib*^−/−^ mice crossed the midline despite an apparent CC hypoplasia (Fig. 5C–D″). Of note, a similar phenotype was observed in adult *Emx1-Scrib*^−/−^ mutant mice using 3D light-sheet microscopy of uDISCO treated brains injected in the caudal cortex with an adenovirus encoding GFP (Fig. 5E,F′) suggesting that ACC is not due to a developmental delay. The deletion of *Scrib* in the *FoxG1*-Cre mice led to ACC along the entire rostrocaudal axis (Fig S3). Since *Scrib* is expressed in glial midline structures (Fig. 1E) that are critical for promoting hemisphere fusion and allowing the CC to cross between hemispheres with the help of axonal guidance signals^36^, we next sought to determine whether CC agenesis observed in *Emx1-Scrib*^−/−^ mutant mice results from mislocalization of glial structures. Immunostaining of P0 coronal sections for GFAP and the axonal marker L1-CAM revealed that early deletion of Scrib resulted in a severe disorganization of the midline glia, with GFAP-positive cells scattered along the dorsal part of the ventricle (FigS4). Parasagittal and horizontal brain sections of the *Scrib*^−/−^ mutant confirmed the presence of callosal defects. We found that the CC length, as determined in Fig. 6A,B (red brackets) was respectively reduced by ~ 40% and ~ 60% in *Emx1-Scrib*^−/−^ (Fig. 6C–E, red brackets) and *FoxG1-Scrib*^−/−^ (Fig S5A–C) mutant mice respectively, compared with control littermates. Horizontal sections from P0 *Emx1-Scrib*^−/−^ and *FoxG1-Scrib*^−/−^ mutant mice confirmed the previously observed ACC and showed a dramatic thinning of the DHC (Fig. 6F–G′, Fig S5D–E′). In contrast, *FoxG1-Scrib* and *Emx1-Scrib* cKOs mutant mice had intact VHC (Fig. 6H–I′, Fig S5F–G′) and AC (arrowheads in Fig. 6C,D, Fig S5A-B). 3D imaging of adult *Emx1-Scrib*^−/−^ mutant mice brains cleared with uDISCO confirmed the absence of the hippocampal commissure in adult animals (Fig. 6J,K).

Together with the previous results, these findings suggest that the commissural deficits observed in absence of *Scrib* are due to a reduction in the number of projecting neurons in layer 2/3 or the cortex, and/or a disruption of the midline glial organization.

### Early loss of *Scrib* induces hyperlocomotion and memory defects

**Figure 7:**
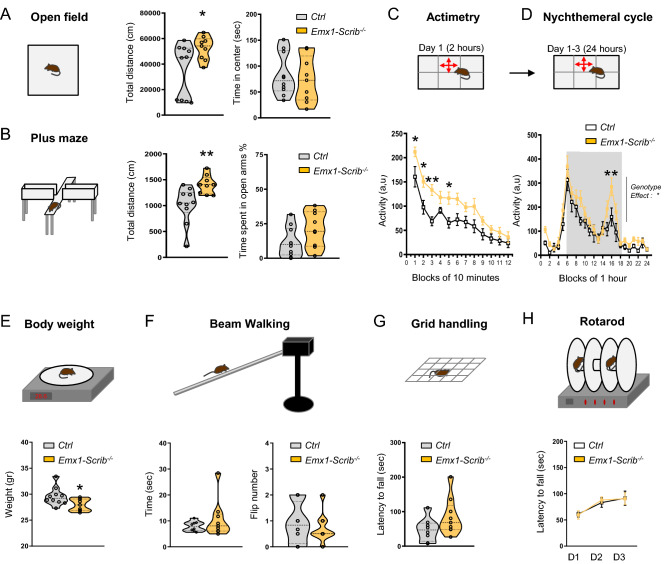
Increased activity without impaired motor coordination in *Emx1-Scrib*^*−/−*^ mice. (**A**) Left panel, open field apparatus. Middle panel, spontaneous locomotor activity measured by the total distance in the open field test. Right panel, time spent in the center. (**B**) Left panel, plus maze apparatus. Middle panel, total distance moved in the plus maze. Right panel, time spent in open arms. (**C**) Upper panel, actimetry test apparatus. Lower panel, distance travelled measured in 10-min intervals across the 120-min test session in a novel home-cage (control: n = 6 mice; *Emx1-Scrib*^−/−^: n = 9 mice). (**D**) Upper panel, nychtemeral cycle test apparatus. Lower panel, distance travelled measured in 1 h intervals across the 24 h test session in a home cage. (**E**) Upper panel, balance apparatus. Lower panel, body weight of 10–11 week-old mice. (**F**) Upper panel, beam walking apparatus. Lower panel, time spent to reach the black box and total flip number. (**G**) Upper panel, Grid handling apparatus. Lower panel, Latency to fall the grid. (**H**) Upper panel, rotarod test apparatus. Lower panel, latency to fall in accelerating (4–40 rpm across 5 min) rotarod test on 3 consecutive days. Data are presented either as violin plots with single data point as a dot or as means ± s.e.m. from 6 to 10 mice per genotype.

**Figure 8:**
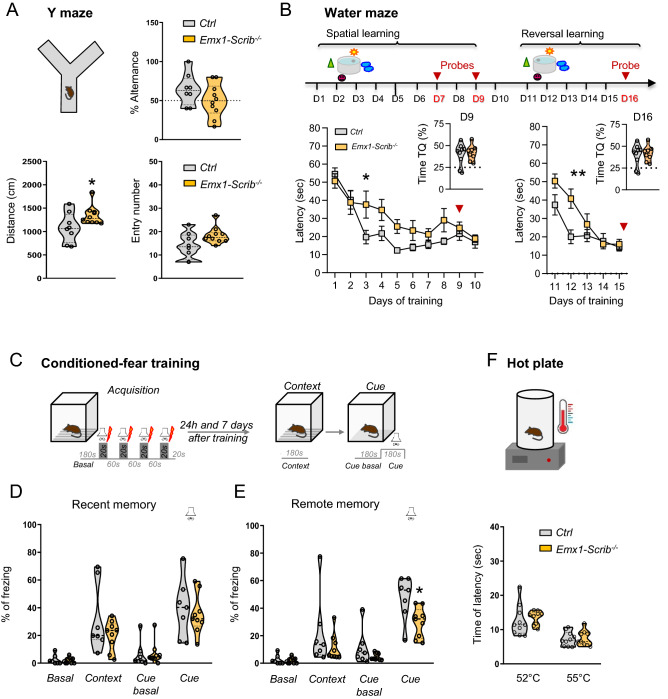
Spatial learning and long term memory deficit in Emx1-Scrib^−/−^ mice. (**A**) Upper panel, Y maze test apparatus and alternance rate percentage in the Y maze test. Lower panel, total distance travelled and total number of entries in the Y maze test (control: n = 8 mice; *Emx1-Scrib*^−/−^: n = 10 mice). (**B**) Upper panel, design of the Morris water maze apparatus task. Lower panel, the learning curve showing escape latency during the spatial and reversal training sessions. Time spent in target quadrant during the probe test at day 9 and day 16 are represented in the right corner of each graph (control: n = 9 mice; *Emx1-Scrib*^−/−^: n = 8 mice). (**C**) Upper panel, design of the fear conditioning apparatus task. (**D,E**) Freezing levels in context and tone fear conditioning. (**D**) Percentage of freezing measured before training (basal), 24 h (recent memory) after training. (**E**) Percentage of Freezing measured before training (basal) and 7 days (remote memory) after training (control: n = 7 mice; *Emx1-Scrib*^−/−^: n = 9 mice). (**F**) Lower, Hot plate apparatus. Time spent to paw withdrawal in the hot plate test at 52 °C and 55 °C (control: n = 10 mice; *Emx1-Scrib*^−/−^: n = 9 mice). Data were represented either as violin plots with single data point as a dot or as mean ± SEM.

To determine the behavioral consequence of the early loss of *Scrib* we next subjected our mutant model to a variety of tests that require sensory-motor, emotional and cognitive integration. Adult *Emx1-Scrib*^−/−^ cKO and their control littermates (10–20 weeks) were submitted to tests for anxiety-, locomotor and exploratory activities. The most remarkable behavior phenotype we observed was the effect on locomotor activity. Compared with their controls, *Emx1-Scrib*^−/−^ mutant mice showed significantly increased activity in the open field (*t* test: *t*_*17*_ = 2.35, *P* < 0.05*; Fig. 7A). The time spent in the center of the arena was not different between genotypes, suggesting that the *Emx1-Scrib*^−/−^ mutant mice has comparable anxiety level to control littermates (*t* test: *t*_*17*_ = 0.4595, *n.s*; Fig. 7A). Anxiety-like behavior was further examined in elevated plus-maze (Fig. 7B), where *Emx1-Scrib*^−/−^ and control mice showed comparable performance, confirming that *Emx1-Scrib*^−/−^ mice have no anxiety-related behaviors (*t* test: *t*_*17*_ = 1.741, *n.s*; Fig. 7B). In addition, we confirmed that the *Emx1-Scrib*^−/−^ mutant mice were significantly more active in elevated plus-maze (*t*-test: *t*_*17*_ = 3.02, *P* < 0.01**; Fig. 7B), in Y maze (*t*-test: *t*_*17*_ = 2.39, *P* < 0.05*; Fig. 8A), and in a new cage as assessed by actimetry during a 2 h period (genotype effect: *F*_1,13_ = 8.757, *P* < 0.05*; Fig. 7C). In addition, *Emx1-Scrib*^−/−^ cKOs mutant mice displayed enhanced locomotor activity in their home cages during nycthemeral activity compared to the control mice, as determined by a 24-h continuous monitoring of locomotor activities (genotype effect: *F*_1,13_ = 6,802, *P* < 0.05*; Fig. 7D). Hyperactivity of *Emx1-Scrib*^−/−^ cKOs mutant mice was associated with a weight loss (*t* test: *t*_*17*_ = 2.412, *P* < 0.05*; Fig. 7E). Notably, *Emx1-Scrib*^−/−^ mice did not display changes in balance, as tested on beam walking (Fig. 7F) and grid handling (Fig. 7G), as well as motor coordination as tested on the accelerating rotarod (Fig. 7H).

Considering that cKOs mice had impaired hippocampal and cortical connectivity, we examined whether the *Emx1-Scrib*^−/−^ mice were impaired in different types of learning and memory tests. In response to novelty, *Emx1-Scrib*^−/−^mutant mice showed a decrease in exploratory activity over time as the context loses its novelty, and ended up no different from control mice, suggesting that this simple form of spatial recognition is preserved in *Emx1-Scrib*^−/−^ cKO mice (time effect: *F*_3,532_ = 50.56, *P* < 0.001***; Fig. 7C). The percentage of alternance was similar in both genotypes suggesting that working memory assessed in Y maze is intact in the mutant mice (*t* test: *t*_*16*_ = 1,404, *n.s*; Fig. 8A). To further probe hippocampus-dependent memory, we next analyzed the effect of early *Scrib* loss on spatial learning and memory using the Morris water maze test. Mice were trained to learn spatial cues around the maze to find a hidden platform under the water during training sessions (training days: *F*_*9,135*_ = *17.59, P* < 0.0001***; Fig. 8B). After training the mice for 15 days and preformed probe tests at day 7, 9 and 16, we observed that *Emx1-Scrib*^−/−^ mice showed significantly longer latency to find the platform during training sessions, demonstrating that the spatial learning is impaired in the mutants (genotype effect: *F*_*1.15*_ = 5.53,* P* < 0.05*; Fig. 8B). Furthermore, in the reversal test the *Emx1-Scrib*^−/−^ mutant mice showed a delay of spatial learning (interaction effect: *F*_*4.60*_ = *4.335, P* < 0.01**; Day2 *t* test: *t*_*13*_ = 3.2, *P* < 0.01**; Fig. 8B). In all probes test, *Emx1-Scrib*^−/−^ mice show normal hippocampus-dependent memory. Importantly, although the *Emx1-Scrib*^−/−^mutant mice were hyperactive, swimming speeds during the probe tests were not different between genotypes. Finally, hippocampus-dependent context and amygdala-dependent tone associative memory was assessed by using classical fear conditioning paradigm in which the animals have to associate environmental cues to an electric shock (Fig. 8C). Two different groups of *Emx1-Scrib*^−/−^ and control mice were re-exposed to the same environment 24 h or 7 days after training, respectively showing the recent (24 h) and the remote (7 days) contextual fear memory (Fig. 8D,E). During the acquisition phase (without shock), all groups of mice displayed a normal freezing behaviour and the first 3 min of acquisition period was considered as the baseline period (basal). Every group exhibited an increase in freezing during the context and tone presentation tested 24 h (test effect: *F*_*1.28*_ = 75.69, *P* < 0.0001***; Fig. 8D) and 7 days after training (test effect: *F*_*1.28*_ = 75.82,* P* < 0.0001***; Fig. 8E). The freezing levels were comparable between genotypes when the memory was tested 24 h after training, showing that the recent contextual and tone fear memories are intact in *Emx1-Scrib*^−/−^ mice (no genotype effect: *F*_*1.56*_ = 1.351, n.s; Fig. 8D). When mice were tested 7 days after training, *Emx1-Scrib*^−/−^ mice froze less than their control littermate during the tone indicating an acceleration of the extinction of the long term cued fear memory (genotype effect: *F*_*1.56*_ = 8.459,* P* < 0.01**; Fig. 8E). Here, *Emx1-Scrib*^−/−^ mutant mice exhibited no significant prolonged latency to paw to licking/jumping in the hot plate test than controls indicating normal nociceptive reactivity (no genotype effect: *F*_*134*_ = 0.37, n.s; Fig. 8F).

Altogether, these results support the idea that *Emx1-Scrib*^−/−^ mutant mice display hyperactivity in novel and familiar environments, which is compatible with the altered psychomotor behavior observed in VRJS patients (see “Discussion”). In addition, the remote memory deficits we observed in this mouse model have not been reported in patients with VRJS and should guide memory evaluation in older patients.

## Discussion

Our present study demonstrates that *Scrib* is essential for embryonic brain development and function. We showed that early deletion of *Scrib* leads to microcephaly and cortex layering defects associated with corpus callosum and hippocampal commissure agenesis. Behavioral analysis showed an increased locomotor activity accompanied by memory defects as a consequence. Our integrative work supports the participation of *Scrib* to various congenital neurodevelopmental deficits.

Both conditional mutants used in this study and with early deletion of *Scrib* (*FoxG1-Scrib*^−/−^ and *Emx1*-Scrib^−/−^ mutant mice) display microcephaly in a range that is comparable to well-established microcephalic mouse models^37^. Microcephaly observed in mutants with embryonic *Scrib* deletion is most likely the result of a combination of disrupted neurogenesis and migration processes, as deficits in any of these critical mechanisms can participate in brain malformation^2^. This multifactor effect of *Scrib* is consistent with its expression profile. It is expressed throughout the cerebral cortex, both in neuronal progenitors and in the radial glia, also supporting a role in migration. In addition, we also observed Scrib enrichment in the VZ and SVZ at the time of neurogenesis that prompted us to analyze the impact of *Scrib* loss on progenitors. We observed in *Emx1*-Scrib^−/−^ mutant mice a decrease in Ki67-positive proliferating cell population (− 12.2% ± 4.9) associated with a reduction of neuronal progenitor populations: Pax6 + cells (− 12.4% ± 6.0), Tbr2 + cells (− 23.3% ± 6.9). We believe that these reductions in progenitor populations, which generate cortical neurons, are responsible for the thinning of the cerebral cortex and associated layering alteration. Finally, we cannot rule out the possibility that it also contributes to asymmetric cell division (ACD), as observed in other systems^6,7^, but we can rule out apotosis deficits.

The other main phenotype we observed after early *Scrib* deletion was the CC and DHC agenesis. These commissural defects may arise from neurogenesis, migration, and/or axonal outgrowth/guidance alterations. *Scrib* inactivation resulted in complete ACC in both rostral and caudal domains in *FoxG1-Scrib*^−/−^ cKOs, while *Emx1-Scrib*^−/−^ cKOs display ACC only in the caudal part. The most likely explanation for this restricted phenotype at the caudal level is that *Emx1* is expressed on a high-caudal to low-rostral gradient^38^. In the rostral telencephalon, *Scrib*-dependent ACC is associated with the formation of abnormal swirls of axons called Probst bundles^36^. Such phenotype is associated with failure of the callosal axons to cross the midline rather than an outgrowth problem towards the midline. Although we cannot completely exclude an autonomous role of *Scrib* in axon guidance as shown during axonal misrouting at the chiasm^39^ and during spinal commissures formation in zebrafish^40^, *Scrib* expression in the midline glia and the disorganization of that structure in *Emx1-Scrib*^−/−^ mutant mice suggest that a non-autonomous *Scrib*-dependent mechanism affects the crossing of the axons of the CC. Mutations in guidance cues expressed by the midline glia typically lead to a Probst bundles phenotype; thus, we can envision that *Scrib* deletion affects midline glial cells maturation and/or guidance cues secretion^41^ as seen in other systems^42,43^. Alternatively, by impacting these astroglial structures that are essential during interhemispheric remodeling^44^, *Scrib* may promote tissue fusion^45,46^, which in turn allow the callosal fibers crossing. Tissue fusion appears as a common theme during neural tube closure^47^ and during interhemispheric remodeling in CC formation^48^. Of note, one third to half of patients with spina bifida also have CC abnormalities^27^ that may explain some behavioral deficits due to improper interhemispheric transfer of information^28^.

By impairing neurogenesis, migration and commissure formation, we show that the absence of *Scrib* during development causes profound defects in the cortical layering and interhemispheric connectivity that underlie cognitive disabilities that are typical of neurodevelopmental disorders and some human syndromes. Edwards and collaborators discriminate two categories of human syndromes: on one hand the ones that display only a microcephalic phenotype and, on the other hand, the ones that do encompass both ACC and microcephaly^3^. *SCRIB* may also fall into the latter category, reflecting a concomitant function for this gene in cortical organization and axonal guidance during development. Our results support the theory that the microcephaly and ACC observed in VRJS patients is the result, at least partially, of *SCRIB* haploinsufficiency.

Behavioral analysis of the *Scrib* cKO mice revealed alterations in psychomotor behavior. Specifically, *Emx1-Scrib*^−/−^ mutant mice had an increased locomotor behavior that is comparable to established hyperactive models^49^. Because of the early and broad expression of *Emx1* in the brain, the pathological origin for the altered locomotor behavior may stem from the cortex and/or the hippocampus, through their connections. Exploration of cognitive performance shows that *Emx1-Scrib*^−/−^ display memory deficit for a cortex-dependent remote-cued fear memory recall. The CC primary function is to integrate motor, sensory, and cognitive activity between the two hemispheres^3^. The DHC provides interhemispheric connections between hippocampi and despite few studies of the function of the DHC, the ability of the hippocampus to communicate effectively with contralateral homologous regions via the DHC may be important for cognitive performance, compounded by the other deficits observed in the mutants. This lack of communication and/or the microcephaly and/or layering defects could be the cause of these cognitive deficits. At the molecular level, it is tempting to speculate that Scrib may control some aspects of brain architecture and behavior, through known partner such as GIT1^50^ or Vangl2^51^. Similar to our model, mice lacking GIT1 have microcephaly^52^ and display a hyperactivity phenotype combined with learning and memory deficits^53^. Like *Emx1-Scrib*^−/−^ mice, *Emx1-Vangl2*^−/−^ display partial hippocampal and CC agenesis (but no microcephaly) that is caused by abnormal axonal outgrowth^54^. However, a conditional deletion of *Vangl2* in postmitotic hippocampal granule cells does not alter spatial memory^55^, highlighting the complexity of such molecular integration at the behavioral level. Additional work from our group support a critical role for Scrib in synaptic dysfunction and human psychiatric disorders^13,17,19^, at least in part, by the regulation of the glutamatergic signaling^18^. Remarkably, the function of this pathway in the process of learning and memory seems to extend toward the invertebrate phylum where *Scrib* is pivotal to the regulation of active forgetting^56^. Future studies exploring these possibilities are needed to define the detailed molecular mechanisms.

Our findings uncover an essential role for *Scrib* in mammalian forebrain development and connectivity, both of which ultimately affect animal behavior. Given that several aspects of the neurological manifestations of VRJS were recapitulated in *Scrib* cKO mice, we suggest that *Scrib* may participate in this syndrome. The minimal common deletion found encompassed 3 genes including *SCRIB* and *PUF60* and displayed most of the cardinal features of VRJS^29^. Although *PUF60* appears to be a major driver of VRJS syndrome^29,57–64^, neurological features were reported in a much lower proportion, indicating that *PUF60* CNVs may not be their sole cause. From the human genetics standpoint, any VRJS-related phenotype due to *SCRIB* loss may prove difficult to observe because most *SCRIB* mutations lead to NTDs so deleterious that they may obscure more “subtle” phenotypes^20–23^. The scarce number of NTD cases carrying *SCRIB* variants or VRJS patients implies the possibility that their true phenotype spectrum may be wider than indicated by publications providing few or no detailed neuropsychiatric evaluation. Patients with spina bifida tend to show altered cognitive abilities with language, memory, motor and psychosocial difficulties^28^. Neurological features for VRJS patients include mild intellectual disability, delay of developmental milestones such as standing upright and walking, delayed speech, feeding issues and generalized seizures^29,30^. All of these features, combined with neuroanatomical features such as microcephaly and CC agenesis can fall under the umbrella of a neurodevelopmental disorders whose affected individuals develop psychomotor deficits reminiscent as those observed in ASDs, ADHD and schizophrenia. Interestingly, a case for a patient with ADHD revealed a chromosomal translocation breakpoint at 8q24.3 has been reported^65^ and this region appears to overlap with ASDs as well^32^. Further studies are warranted in order to determine whether other behavioral phenotypes such as seizure susceptibility or deficits of attention/impulsivity, are also recapitulated in *Scrib*^−/−^ cKOs. Our study demonstrates that *Scrib* mutant mice can provide an entry point to study its forebrain contribution in neuroanatomical and behavioral deficits observed in NTD and VRJS patients. Future investigation using both heterozygous *Scrib* KO and *Puf60* KO mice (alone or in combination) as a model is warranted to address their respective contribution and potential interaction in this syndrome at a more systemic level.

## Materials and methods

### Animal care and use

All mice were housed in the animal facility of the Neurocentre Magendie, in polypropylene cages under controlled conditions (at 23 ± 1 °C, with lights on from 7:00 A.M. to 7:00 P.M.). Food and water were available ad libitum. For timed pregnancy, the morning in detection of vaginal plug was designated as embryonic day E0.5. This study was performed according to the European Communities Council Directives (2010/63/EU) and has been approved by the Animal Care and Use Committee (Bordeaux) under the numbers 5012016-A and 5012015-A. This study was carried out in compliance with the ARRIVE guidelines.

### Generation of brain-specific *Scrib* conditional knock-outs

Scrib spontaneous mouse mutant *Circletail* causes severe brain and neural tube damages that result in neonatal lethality^8^, precluding the analysis of the role of *Scrib* during forebrain development^17^. In order to circumvent this issue, we have applied a conditional gene-targeting strategy to inactivate *Scrib* at different developmental stages and in different cellular types in the brain. See Supplementary material and methods for details.

### Histology, in situ hybridization analysis and immunofluorescence

For histology, heads (E16.5 embryos) or brains (P0 new-born) were harvested and fixed in Bouin’s or Carnoy’s fixative (Electron Microscopy Sciences) overnight as previously described^66^. Samples were dehydrated in ethanol, paraffin-embedded, and sectioned (20 µm) coronally, horizontally or sagitally. Sections were collected onto Superfrost plus Gold slides (Thermo Scientific), stained with hematoxylin and mounted with Entellan (Millipore). The brains sections were examined using Leica MZ-16 stereomicroscope, imaged in the Bordeaux Imaging Center (http://www.bic.u-bordeaux.fr) using the NanoZoomer 2.0-HT slide scanner and analyzed using the Hamamatsu NDP viewer software (Hamamatsu). For in-situ hybridization, E16.5 and E17.5 embryos brains were dissected out, transferred in OCT solution and placed into the dry ice for storage at − 80 °C before sectioning. In situ hybridization was performed using previously validated digoxigenin-labeled cRNA probes^17^. 16 µm coronal embryonic brain sections of were postfixed in 4% paraformaldehyde/0.2% glutaraldehyde for 10 min at room temperature (RT), bleached with 6% H_2_O_2_, digested in Proteinase K (5 µg/ml in PBS) for 2.5 min and postfixed in 4% paraformaldehyde/0.2% glutaraldehyde for 10 min at RT. Then, slides were acetylated into freshly prepared 0.1 M triethanolamine/PBS, pH 8, for 10 min at room temperature; 0.25% acetic anhydride acid was added for the last 5 min. Between each step, slides were rinsed with PBS. All subsequent steps were performed as previously described in^17^. Images were acquired with Nanozoomer (Hamamatsu). For immunofluorescence staining (IF), P0 pups were anesthetized with pentobarbital, then perfused transcardially with 4% paraformaldehyde (PFA) in PB buffer (0.1% PBS, 0.9% NaCl, PH 7.4). Dissected brains were postfixed in 4% PFA overnight at 4 °C, infused in 30% sucrose in PB overnight and embedded in O.C.T (Sakura Finetek). The OCT-embedded brains were cryosectioned coronally at a thickness of 20 μm, mounted on Superfrost plus slides (VWR), washed with PBS, and immunostaining was performed. Sections were hydrated with PBS, permeabilized with 0.2% Triton-X100/PBS (PBS-T), blocked using 10% Normal Goat Serum (NGS) or Normal Donkey Serum (NDS) and incubated for 1 h at RT or overnight at 4 °C with the following primary antibodies in 1% serum: rabbit anti-GFAP (DAKO; #Z0334;1:1000), rat anti-L1-CAM (Millipore; #MAB5272; 1:1000), rat anti-Ctip2 (Abcam; #ab18465, 1:500), mouse anti-Satb2 (Abcam; #ab51502, 1:100), rabbit anti-Tbr1 (Millipore; #AB9616; 1:500), rabbit anti-Cux1 (Santa Cruz Biotechnology; #sc-13024; 1:100), rabbit anti-Tbr2 (Abcam; #ab183991; 1:1000), rabbit anti-Pax6 (Biolegend; #901301; 1:1000), rabbit anti-Ki67 (Cell Signaling Technology; #9129; 1:400), anti-cleaved Caspase-3 (Asp175) (Cell Signaling Technology; #9661; 1:1000), goat anti-Scrib (Santa Cruz; #sc-11049; 1:200 to 1:1000), rabbit anti-Scrib (homemade AbMM468; 1:300)^51^. Samples were washed three times in PBS-T and were incubated for 1 h with the secondary antibodies Alexa Fluor 488 or 546 or 594 Goat Anti-Mouse/Rat/Rabbit IgG or Donkey Anti-Goat IgG (Jackson Immunoresearch or Life Technologies; 1:500 to 1:2000) and then with DAPI (Life Technologies; 1:20,000) for 30 min. Finally, samples were washed three times in PBS and mounted with Prolong Gold anti fading reagent (Invitrogen). Immunostained sections were imaged at similar brain region using a Zeiss Axio Imager Z1 microscope and Axio Vision (Version 4.7) imaging analysis software (Carl Zeiss). All images were processed with Photoshop CS5 software (Adobe) and ImageJ software (http://imagej.nih.gov).

### UDisco

Eight to 10-week old *Emx1-Scrib*^−/−^ mice received unilateral stereotaxic microinjections of a AAV (0.5/1 µl at 300 nl per min) expressing GFP under the control of the promoter hSyn1 (AAV9-hSyn1-GFP-2A-GFP-f, titer 4.8 × 10^12^ GC/ml) in the sensory-motor cortex region (anteroposterior [Y] − 2 mm from Bregma, mediolateral [X] ± 1.2 mm, dorsoventral [Z] – 0.5 mm) or expressing mCherry under the control of the CamKII promoter (AAV2/9-CamKII(0,4)-mcherry-WPRE, Vector Biolabs, titer 1.2 × 1013 GC/ml) in the CA3 region of hippocampus (anteroposterior [Y] − 2 mm from Bregma, mediolateral [X] ± 3 mm, dorsoventral [Z] − 2,5 mm). Four weeks after surgery, the animals were perfused transcardially with PB followed by 4% PFA in PBS; the brains were removed and postfixed in 4% PFA for 24 h at 4 °C and maintained in PBS. The entire brains were cleared using the uDISCO technique as described^67^. The ultramicroscopy was performed using the system from LaVision BioTec (Bielefeld, Germany) equipped with a Fianium white laser, a sCMOS Andor camera, and a 0.5 NA 2X objective with a deeping lens. A zoom from 0.63 to 6.3 could be applied. Images and 3D reconstruction were analyzed with Image J and Imaris software.

### In-utero electroporation and tissue processing

In utero electroporation experiments were performed according to protocols previously described^68^. The Animal Care and Use Committee (Bordeaux) has approved the experimental procedure under the number 5012015-A. Pregnant Swiss CD-1 mice were anesthetized using 4% isoflurane in an anesthesia induction chamber, maintained with 2% isoflurane with an anesthetic mask and injected before surgery with buprenorphine. Mice were subjected to abdominal incision; uterine horns were exposed and E14.5 embryos were placed on humidified gauze pads. Plasmid DNA was purified on Qiagen columns (EndoFree Plasmid Maxi Kit), resuspended in sterile endotoxin-free buffers (Qiagen) and mixed with Fast Green (Sigma). mCherry plasmid, together with pSuper or validated pSuper-Scrib shRNA construct (0.5 μg/μl)^17^ were microinjected at a 1:3 ratio into the lateral ventricles of embryos. Five current pulses (50 ms pulse/950 ms interval; 35–36 V) were delivered across the heads of the embryos using 7 mm electrodes (Tweezertrode 450165, Harvard Apparatus) connected to an electroporator (ECM830, BTX). Surgical procedure was completed with suture of the abdomen wall and skin. E18.5 embryos or P0 pups were processed for tissue analysis and immunostaining as described in the histology section. Subregions of the cerebral cortex (VZ/SVZ, IZ, LL and UL) were identified based on cell density using DAPI staining (Life Technologies; 1:20,000). For each condition, sections from three embryos obtained from three separate litters were quantified. Quantification of mCherry-positive cells was performed using the cell counter plugin for ImageJ (http://rsbweb.nih.gov/ij/plugins/cell-counter.html). Data are given as a percentage of the total of cells positive for mCherry in each cortical subregion (mean ± SD).

### Behavioral testing

Behavioral experiments were conducted with *Emx1-Scrib*^−/−^ cKOs and their control littermate male mice of 10–11 weeks of age at the start of behavioral tests. All behavioral experiments were performed during the light phase (between 9:00 A.M. and 7.00 P.M.) of a 12 h light/dark cycle, under conditions of dim light and low noise. One week before starting the experiments, mice were housed in individual cage. Several cohorts of animals and multiple behavioral tests were used. Whenever possible, naïve animals were employed for behavioral testing; when the same cohort was used for multiple tests, the most stressful assays were performed last, to minimize between-test interference. To look for behavioral abnormalities mice were tested in activity cages (to measure locomotor activity); in elevated plus maze, open field and Y-maze (to measure exploratory activity and anxiety-like behavior); in rotarod, hot plate, beam walking and grid handling test (to measure sensory-motor activity); in the Morris water maze to test the spatial memory and in the fear conditioning test maze (to measure early and remote contextual end cued memory performance). All experimental apparatuses were cleaned with hydroalcoholic solution (Phagospray-DM) between subjects to remove odor residuals. See Supplementary material and methods for details.

### Quantification, statistical analysis and data representation

Details of statistical analyses and n values are provided in the figure legends subsections referring to individual assays. Statistical analyses were carried out using the GraphPad Prism statistical package (GraphPad). Normality of distribution and homogeneity of variance were validated and unpaired Student's two-tailed t tests for two data sets were used to compare groups with similar variance and are indicated along the P values in figures. P ≤ 0.05 was considered as statistically significant. Statistics were derived from at least three independent experiments and not from technical replicates. For behavior analyses, two-way ANOVA testing or repeated measure ANOVA was used for the evaluation of the effect of genotype and time in actimetry, motor activation, rotarod, water maze and fear conditioning behavioral test. The Bonferroni posthoc test was used when appropriate. The Student's t test was used for comparing genotype in other behavior tests. Whenever adequate, individual data points were reported as scatterplots to provide full information about the variability of data sets.

## Supplementary Information

Supplementary Information 1.

Supplementary Information 2.
