# Supplementary material for: Early loss of Scribble affects cortical development, interhemispheric connectivity and psychomotor activity
Source: Sci Rep. 2021 Apr 27;11:9106. doi: 10.1038/s41598-021-88147-1 (PMC8079449; doi:10.1038/s41598-021-88147-1)
Supplement: Supplementary file 1 — Supplementary Information 1. [file 41598_2021_88147_MOESM1_ESM.pdf]

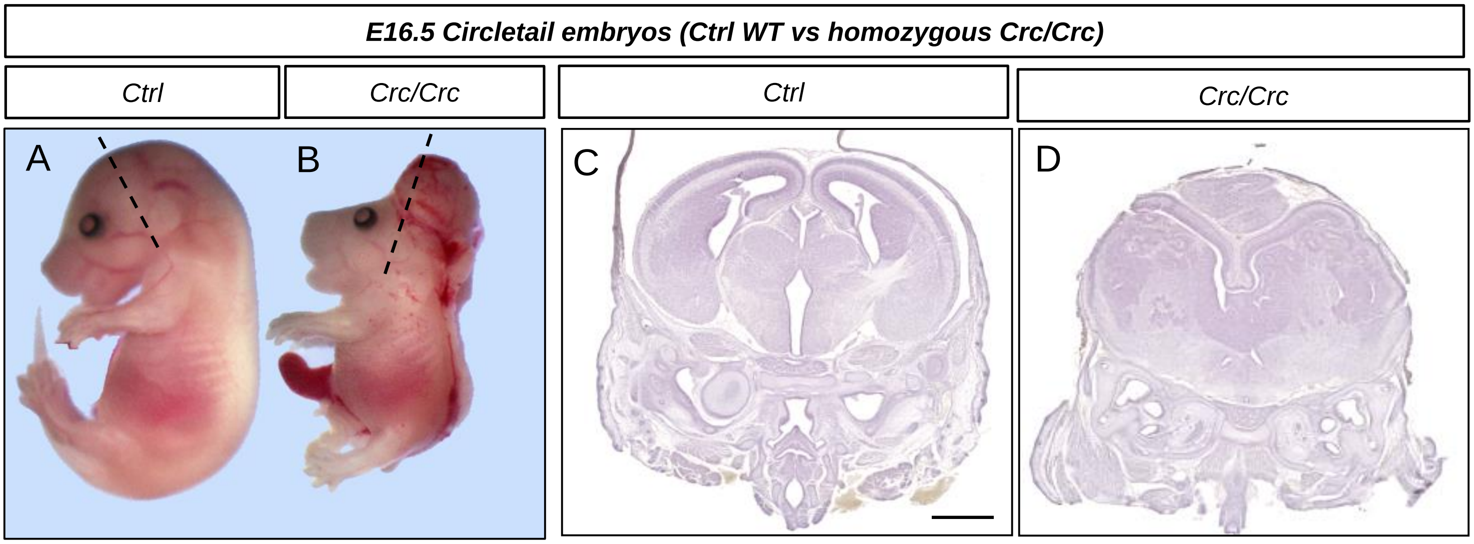
E16.5 Circletail embryos (Ctrl WT vs homozygous Crc/Crc)
Ctrl
Crc/Crc
Ctrl
Crc/Crc
A
B
C
D

Figure S1

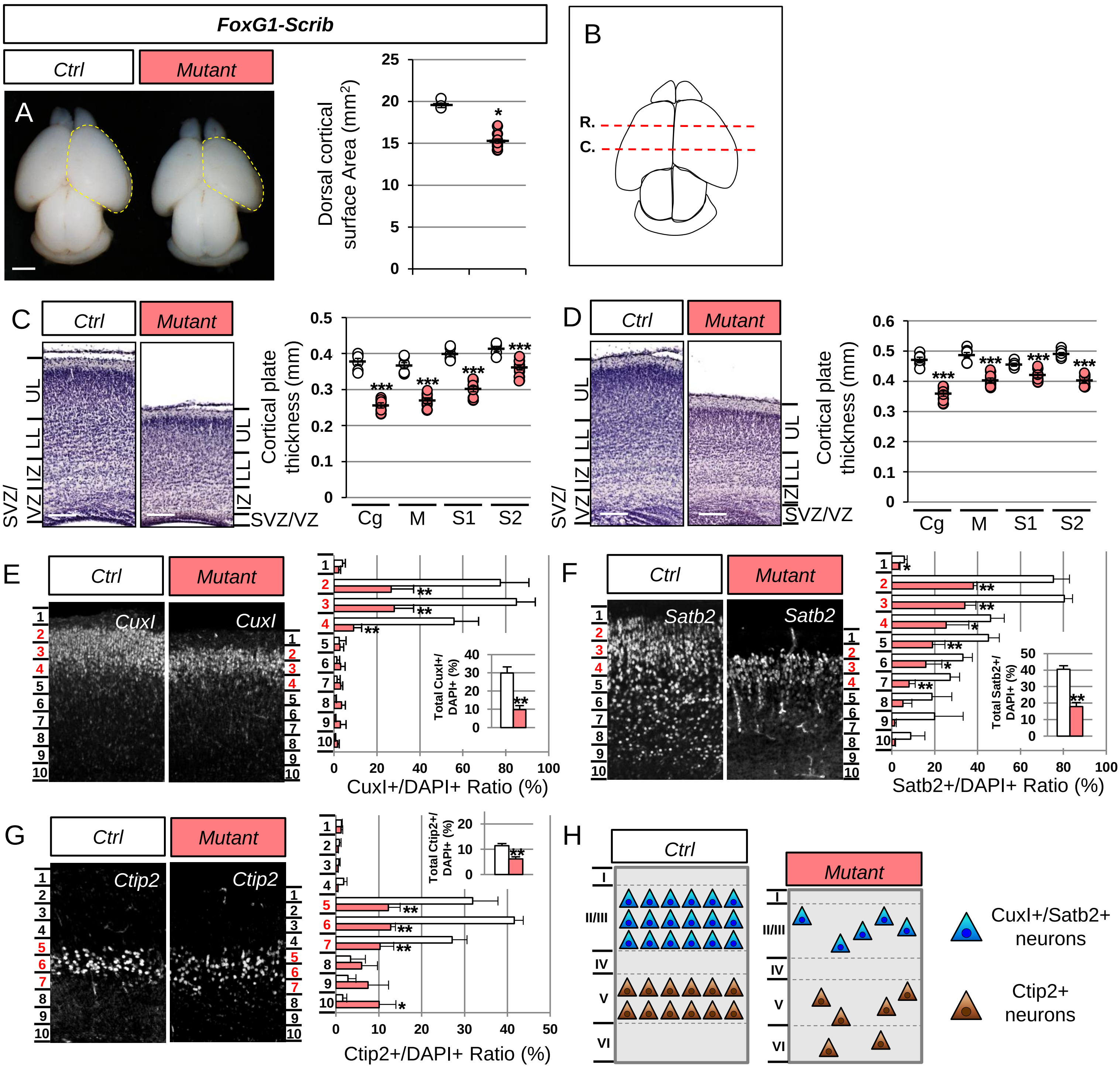
FoxG1-Scrib
Ctrl
Mutant
A
B
R.
C.
Dorsal cortical surface Area (mm2)
C
D
UL
LL
IZ
SVZ/VZ
Cortical plate thickness (mm)
Cg
M
S1
S2
E
CuxI
CuxI+/DAPI+ Ratio (%)
Total CuxI+/DAPI+ (%)
F
Satb2
Satb2+/DAPI+ Ratio (%)
Total Satb2+/DAPI+ (%)
G
Ctip2
Ctip2+/DAPI+ Ratio (%)
Total Ctip2+/DAPI+ (%)
H
I
II/III
IV
V
VI
CuxI+/Satb2+ neurons
Ctip2+ neurons

Figure S2

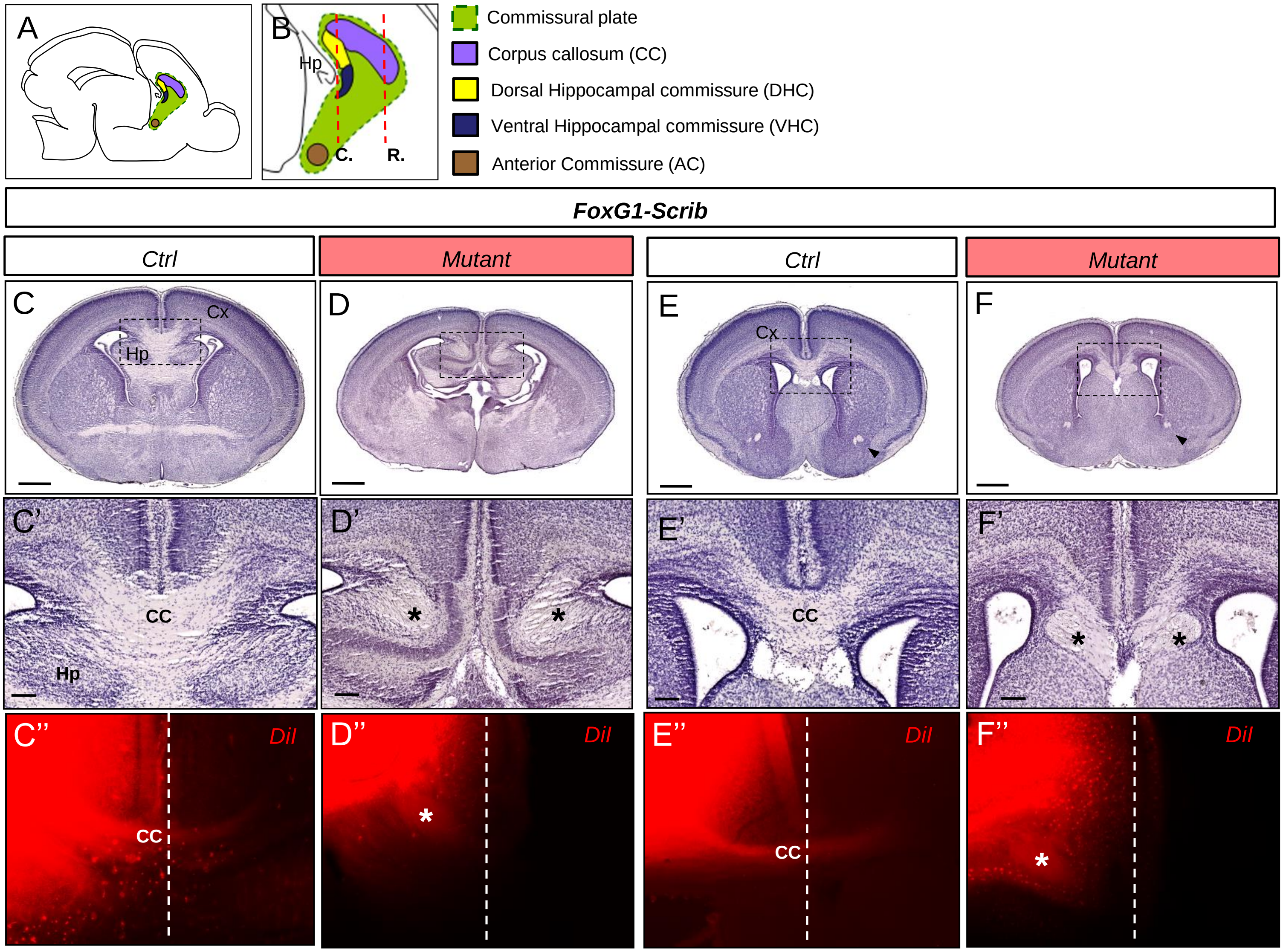
A
B
Hp
C.
R.
Commissural plate
Corpus callosum (CC)
Dorsal Hippocampal commissure (DHC)
Ventral Hippocampal commissure (VHC)
Anterior Commissure (AC)
FoxG1-Scrib
Ctrl
Mutant
Ctrl
Mutant
C
Cx
Hp
D
E
Cx
F
C'
CC
Hp
D'
*
*
E'
CC
F'
*
*
C''
DiI
CC
D''
DiI
*
E''
DiI
CC
F''
DiI
*

Figure S3

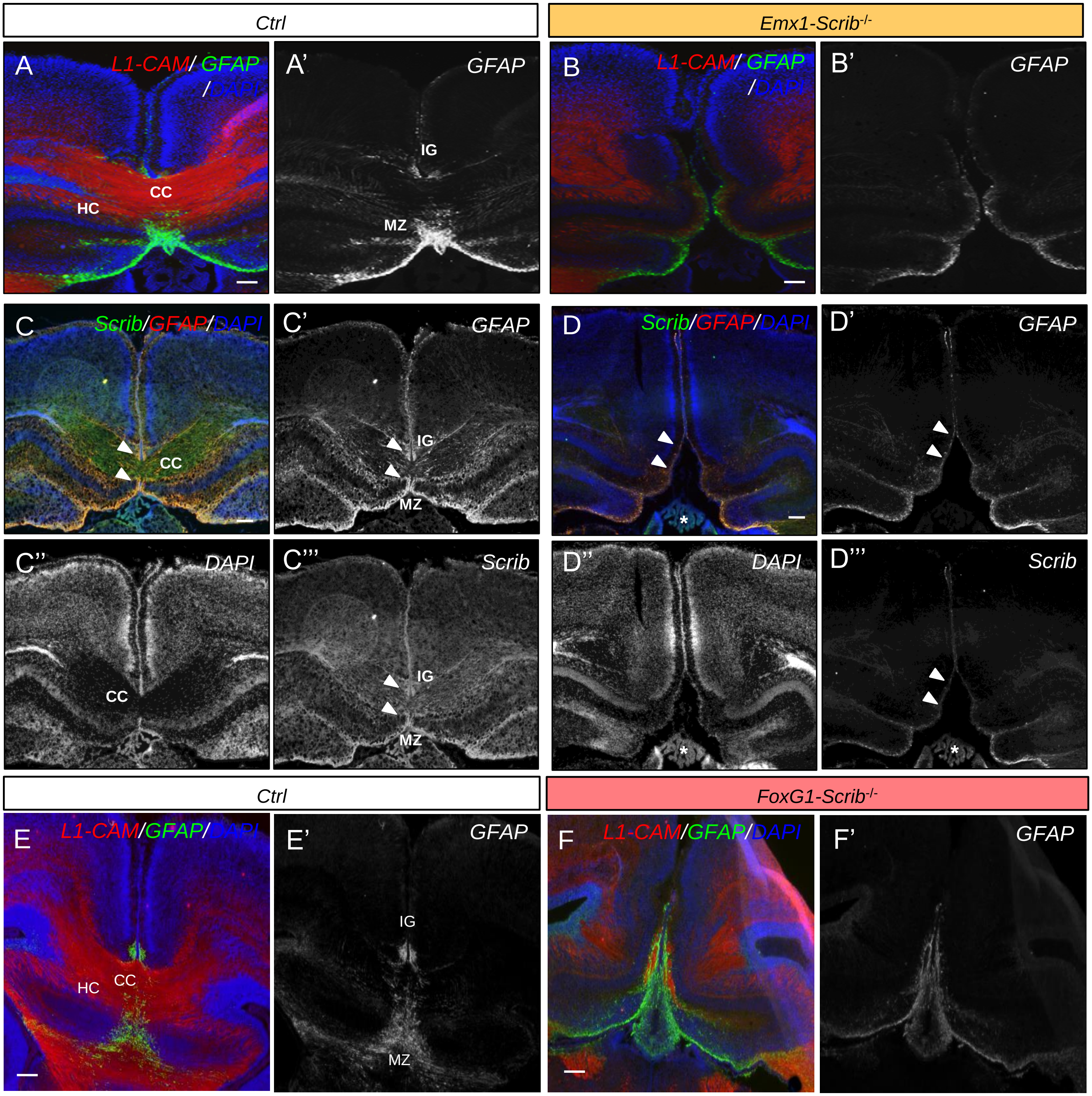

Ctrl
Emx1-Scrib-/-
A
L1-CAM/ GFAP /DAPI
HC
CC
A'
GFAP
IG
MZ
B
L1-CAM/ GFAP /DAPI
B'
GFAP
C
Scrib/GFAP/DAPI
CC
C'
GFAP
IG
MZ
D
Scrib/GFAP/DAPI
*
D'
GFAP
C''
DAPI
CC
C'''
Scrib
IG
MZ
D''
DAPI
*
D'''
Scrib
*
Ctrl
FoxG1-Scrib-/-
E
L1-CAM/GFAP/DAPI
HC
CC
E'
GFAP
IG
MZ
F
L1-CAM/GFAP/DAPI
F'
GFAP

Figure S4

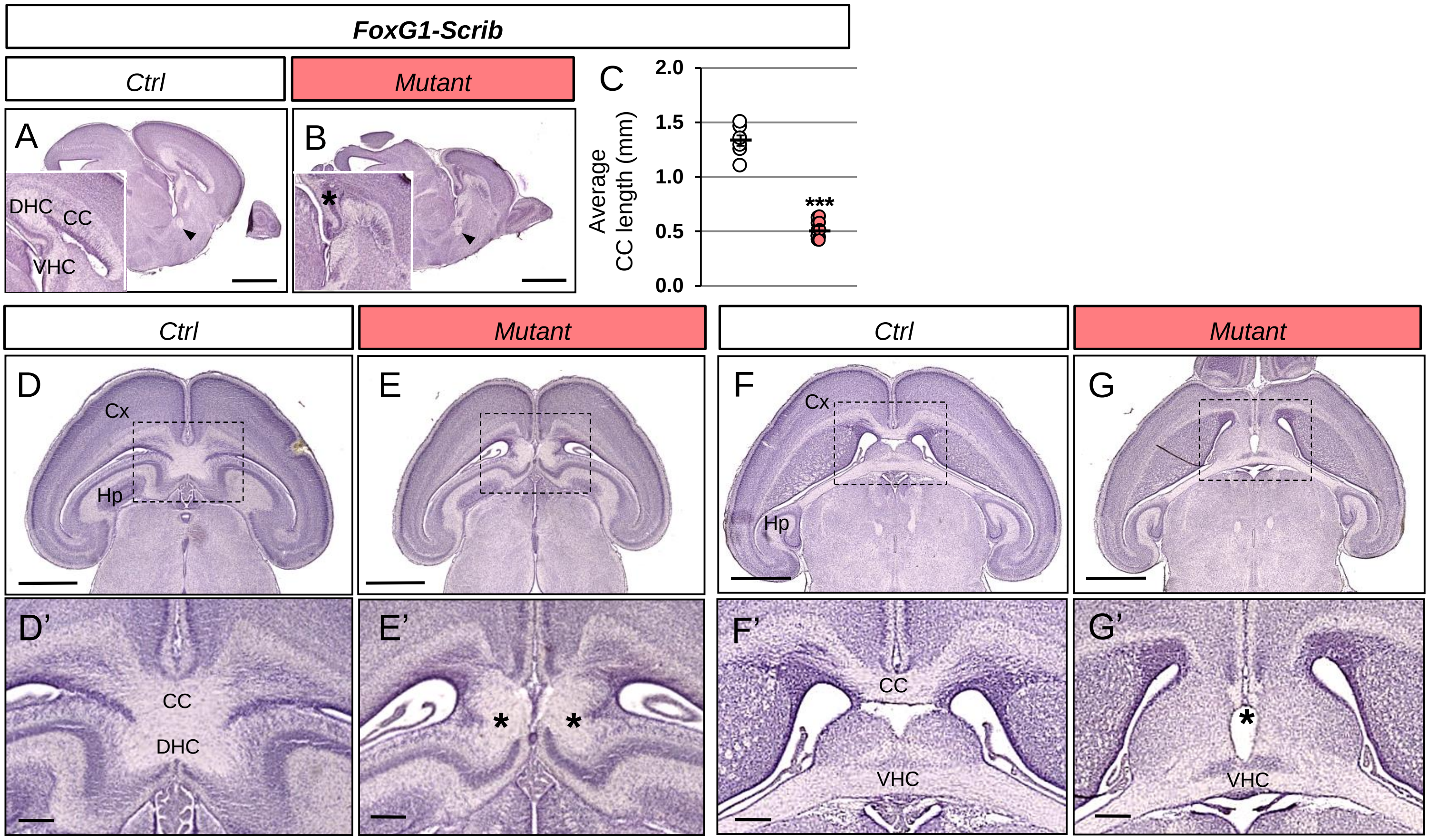
FoxG1-Scrib
Ctrl
Mutant
A
DHC
CC
VHC
B
*
C
2.0
1.5
1.0
0.5
0.0
Average CC length (mm)
***
Ctrl
Mutant
Ctrl
Mutant
D
Cx
Hp
E
F
Cx
Hp
G
D'
CC
DHC
E'
* *
F'
CC
VHC
G'
*
VHC

Figure S5

Full scans of Western Blots shown in Figure 2C

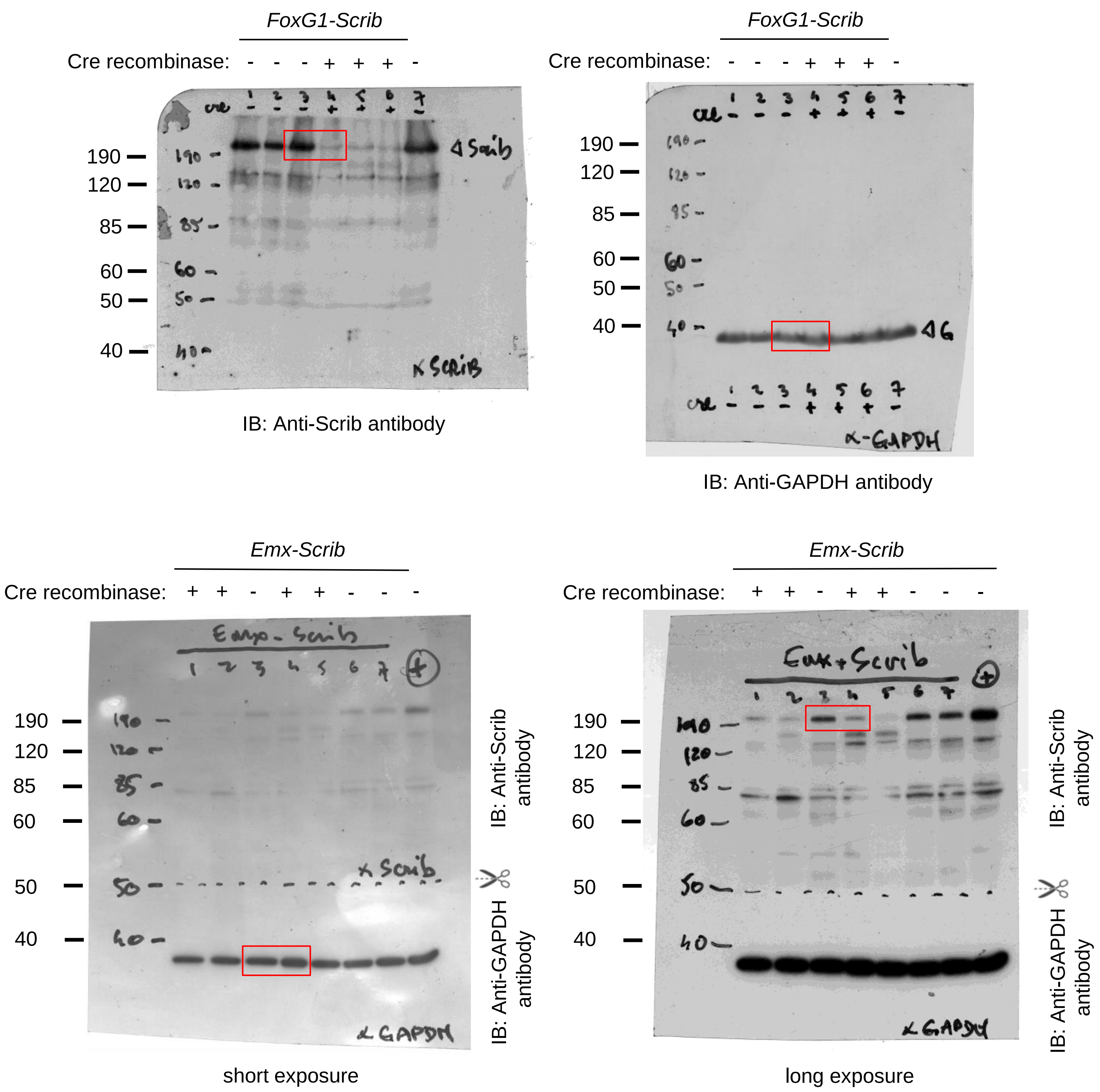

Figure S6
