# Supplementary material for: Early loss of Scribble affects cortical development, interhemispheric connectivity and psychomotor activity
Source: Sci Rep. 2021 Apr 27;11:9106. doi: 10.1038/s41598-021-88147-1 (PMC8079449; doi:10.1038/s41598-021-88147-1)
Supplement: Supplementary file 2 — Supplementary Information 2. [file 41598_2021_88147_MOESM2_ESM.docx]

**Full Title (12/20 words): *Early loss of Scribble affects cortical development, interhemispheric connectivity and psychomotor activity.***

Jerome Ezan^1^, Maité M. Moreau^1^, Tamrat M. Mamo^1^, Miki Shimbo^1^, Maureen Decroo^1^, Melanie Richter^2^, Ronan Peyroutou^1^, Rivka Rachel^3^, Fadel Tissir^4^ , Froylan Calderon de Anda^2^, Nathalie Sans^1^ and Mireille Montcouquiol^1^

1. Univ. Bordeaux, INSERM, Neurocentre Magendie, U1215, F-33077 Bordeaux, France

2. Germany Center for Molecular Neurobiology Hamburg (ZMNH), Research Group Neuronal Development, University Medical Center Hamburg-Eppendorf, Hamburg, Germany.

3. Neurobiology-Neurodegeneration and Repair Laboratory, National Eye Institute, NIH, Bethesda, Maryland 20892, USA.

4. University of Louvain, Institute of Neuroscience, Developmental Neurobiology Group, Avenue Mounier 73, Box B1.73.16, 1200 Brussels, Belgium.

**Corresponding authors**: Dr. Mireille Montcouquiol ([mireille.montcouquiol@inserm.fr](mailto:mireille.montcouquiol@inserm.fr)) and Dr. Jerome Ezan ([jerome.ezan@inserm.fr](mailto:jerome.ezan@inserm.fr))

**Key words**: Scrib; Verheij/8q24.3 deletion syndrome; planar cell polarity PCP; neurodevelopmental disorders; microcephaly; corpus callosum

# Number of figures: 8

**Total word count:** 4383/4500 words

# Supplementary materials and methods

## Generation of brain-specific *Scrib* conditional knock-outs

*FoxG1*-Cre, *Emx1*-Cre and the Cre reporter mouse strain B6.Cg-*Gt(Rosa)26Sor^tm6(CAG-ZsGreen1)Hze^*/J (Ai6) mice ^1^ were purchased from The Jackson Laboratory (Bar Harbor, ME). *Scrib^fl/fl^* floxed mice were generated previously in collaboration with Neal Copeland, Nancy Jenkins and Rivka Rachel at NIC/NIH ^2^. *Scrib* mouse gene contains 38 exons that are translated into a full-length 180 kDa protein. In *Scrib^fl/fl^* floxed mice, lox-P sequences are inserted before exon 2 and after exon 8 ^3^. When crossed with a mouse line expressing the Cre recombinase, all the exons between 2 and 8 are excised, leading to the loss of the full length protein. Heterozygous rodents were intercrossed to generate homozygous and wildtype littermates as previously published ^4^*. Scrib^fl/fl^* floxed mice were either crossed with *FoxG1*-Cre or *Emx1*-Cre mice to obtain compound mice, respectively referred to as *FoxG1-Scrib*^-/-^ and *Emx1-Scrib*^-/-^ mutant mice with early excision in different cellular types in the brain. All Scrib^-/-^ cKO mice are viable, fertile and do not display any gross physical abnormalities. Cre-mediated recombination in *FoxG1*-Cre mice starts as early as E8.5 and occurs mostly in all cells from both dorsal and ventral telencephalon ^5^. This includes progenitors from neuroepithelium that will specify various neuronal and glial cells from the neocortex and hippocampus, but also from the ganglionic eminence that will give rise to GABAergic interneurons. In *Emx1*-cre mice, the Cre recombinase is expressed later as compared with *FoxG1*-Cre mice, starting as early as E10.5 and only in the dorsal telencephalon ^6^. Emx1 pattern of expression lead to the excision of *Scrib* in the vast majority of the neurons of the neocortex and hippocampus, in glial cells of the pallium, but not in the GABAergic interneurons. Genotyping was performed as described previously by PCR using the following primers: F (5’-gcacactgggtatcatggcta-3’), R1 (5’-gcaatctccagagccttacaga-3’), R2 (5’-cccttggaaacctacatcccaa-3’) ^3^. Wild-type (WT), floxed, deleted *Scrib* alleles were distinguished by the following amplified products: for WT band (F+R1; 437 bp), flox band (F+R1; 541 bp) and cKO band (F+R2; 193 bp) **(Fig 2A)**. Cre genotyping was performed using the following primers: F (5’-cggcatggtgcaagttgaata-3’), R (5’-gcgatcgctattttccatgag-3’), resulting in a 300 bp band. PCR products were analyzed on a Labchip GX microfluidic electrophoresis system (Perkin-Elmer) using the DNA5k kit and visualized by a virtual gel image. Cre minus littermates showed no detectable phenotype and were used as controls. The body weight of Emx1-Scrib^-/-^ was slightly lighter than their littermate controls (not shown). It is worth noting that none of the *Scrib*^-/-^ mutants were homozygous for the knock-in *Emx1*-Cre allele (i.e., all were obligate Cre heterozygotes), thus avoiding potential confounds related to *Emx1* loss-of-function. The Cre reporter mouse strain B6.Cg-*Gt(Rosa)26Sor^tm6(CAG-ZsGreen1)Hze^*/J (Ai6) ^1^ which expresses ZsGreen1 from the *Rosa26* locus, was used for evaluation of Emx1-Cre-induced recombination efficiency. *Emx1-Scrib*^-/-^ mice were crossed with Ai6 reporter mice, resulting in enhanced green fluorescent protein (ZsGreen1) expression following Cre-mediated recombination selectively in Emx1-expressing cells within the cortex, hippocampus, fimbria and fibers within the caudate putamen **(Fig 2G-H)**. In contrast, the striatum (ZsGreen1-negative) lacked Cre activity, consistent with Emx1 expression pattern in the dorsal telencephalon.

**Quantitative analysis of laminar position in the cerebral cortex**

Sections chosen for analysis were matched along the rostral-caudal axis and observed at the caudal level for every sample (where ACC is observed in Emx1-Scrib^-/-^ cKO brains). Immunofluorescence images were converted to gray values and normalized to background staining. Regions of interest (ROIs) with a fixed width (but of variable length, corresponding to the thickness of the cortex) were positioned in the motor cortex region with the long axis perpendicular to the pial surface. Each ROI was subdivided into ten equal bins from the pia (bin 1) to the outer border of the intermediate zone (bin 10) to assess CPN distribution across the layers of the cortex. In such arrangement, layers II-III (Cux1-positive) were corresponding to bins 2-4 while layer V (Ctip2-positive) was corresponding to bins 5-7 (highlighted in red in every figure). Slides were evaluated blindly to the genotype and the number and distribution of CuxI-, Satb2-, Ctip2- and DAPI-labeled cells in each zone was determined manually using the cell counter plugin for ImageJ (http://rsbweb.nih.gov/ij/plugins/cell-counter.html). In order to take into account a possible effect of Scrib deletion on total cell number in the cortex, data are given as a ratio (in percent) of the total of cells positive for each marker to DAPI-positive cells in each bin (mean ± SEM). Analysis was performed using at least 3 independent experiments (3 to 4 brains per genotype were quantified). The effect of the genotype on the distribution of cells within the bins was assessed using the Student test (*t test*).

## Callosal Axon tracing

Corticocortical tract tracing was performed as previously described ^7^ using fluorescent carbocyanine dye (1, 1′-Dioctadecyl-3, 3, 3′, 3′-Tetramethylindocarbocyanine Perchlorate; DiI, Invitrogen). Brains were dissected at P0 and fixed in 4% PFA. A single DiI crystal was inserted in the dorsomedial cortex and allowed to diffuse for 1 month. Coronal vibratome sections of 150 μm thickness were cut on a vibratome, mounted on slides and imaged as described in the previous paragraph.

**Western blot**

Cerebral cortices of P0 cKO pups and their control littermates were homogenized in RIPA buffer (10 mM Tris-Cl (pH 8.0), 1 mM EDTA, 0.5 mM EGTA, 1% Triton X-100, 0.1% sodium deoxycholate, 0.1% SDS, 140 mM NaCl) supplemented with protease inhibitor cocktail (Complete; Roche). Protein concentration was determined using Pierce BCA Protein assay kit (Thermo Scientific). Equal amounts of protein were diluted in sample buffer, separated by sodium dodecyl sulfate–polyacrylamide gel electrophoresis (SDS-PAGE), and visualized using the enhanced chemiluminescence (ECL) as previously described ^4^. Briefly, protein extracts were separated on an 8% SDS-PAGE and transferred overnight to polyvinylidene difluoride membranes (Millipore). Membranes were blocked with 5% nonfat milk in 1×Tris-buffered saline pH 7.4; 0.05% Tween-20 (TBS-T) for 30 min at room temperature (RT) and were probed with homemade rabbit anti-Scrib (MM468; 1:500) ^8^ and mouse anti-GAPDH (Millipore; 1:1000) for 1hr RT. Membranes were incubated with secondary antibodies (GE Healthcare UK; donkey anti-rabbit or anti-mouse IgG conjugated to horseradish peroxidase, 1:5000) in 1% nonfat milk in TBS-T for 1hr RT. Each incubation step was followed by 3 washes with TBS-T for 10 min and immunoreactive signals were detected using Pierce ECL substrate (Thermo Scientific). Band intensity was analyzed by densitometry with the ImageJ software (http://imagej.nih.gov) and quantified as a percentage of control band intensity using a representative of at least three independent experiments. Unprocessed original scans of blots are shown in supplementary Figure S6.

## *In-utero* electroporation and tissue processing

*In utero* electroporation experiments were performed according to protocols previously described ^9^. The Animal Care and Use Committee (Bordeaux) has approved the experimental procedure under the number 5012015-A. Pregnant Swiss CD-1 mice were anesthetized using 4 % isoflurane in an anesthesia induction chamber, maintained with 2 % isoflurane with an anesthetic mask and injected before surgery with buprenorphine. Mice were subjected to abdominal incision; uterine horns were exposed and E14.5 embryos were placed on humidified gauze pads. Plasmid DNA was purified on Qiagen columns (EndoFree Plasmid Maxi Kit), resuspended in sterile endotoxin-free buffers (Qiagen) and mixed with Fast Green (Sigma). mCherry plasmid, together with pSuper or validated pSuper-Scrib shRNA construct (0.5 μg/μl) ^10^ were microinjected at a 1:3 ratio into the lateral ventricles of embryos. Five current pulses (50 ms pulse / 950 ms interval; 35–36 V) were delivered across the heads of the embryos using 7 mm electrodes (Tweezertrode 450165, Harvard Apparatus) connected to an electroporator (ECM830, BTX). Surgical procedure was completed with suture of the abdomen wall and skin. E18.5 embryos or P0 pups were processed for tissue analysis and immunostaining as described in the histology section. Subregions of the cerebral cortex (VZ/SVZ, IZ, LL and UL) were identified based on cell density using DAPI staining (Life Technologies; 1:20000). For each condition, sections from three embryos obtained from three separate litters were quantified. Quantification of mCherry-positive cells was performed using the cell counter plugin for ImageJ (http://rsbweb.nih.gov/ij/plugins/cell-counter.html). Data are given as a percentage of the total of cells positive for mCherry in each cortical subregion (mean ± SD).

**Behavioral testing**

*Plus maze, Open field, Y maze and locomotor activity.* Elevated plus maze, Open field and Y-maze experiments were performed as described previously ^3,10^. Locomotor activity in response to novelty and daily rhythm of activity experiments were performed as described previously ^10^.

*Rotarod.* Mice performance on the rotarod, which accelerates from 4 to 40 rpm in 5 minutes, was evaluated for 5 trials per session on three consecutive days. A resting time of 15 minutes was allowed between each trial. The end of a trial was considered when mice were falling off the rod. Latency to fall was recorded for each trial. The average latency was calculated for each testing day.

*Hot plate.* Animals were placed individually on a hot plate with the temperature adjusted to 52°C and 55°C.Response latency (sec) to jump or lick the hind paws was measured. The cut off time was taken as 30 seconds to avoid risk of thermal injury to the skin.

*Beam walking*. During three successive days, mice were placed on one end of a wood beam (180 cm in length with a 2 or 1 cm square cross-section) at a height of 50 cm above a container with soft bedding and the time required to reach an enclosed safety box at the other end (80 cm away) is measured. On training day 1, beam was place horizontally and each mouse was trained to traverse the 2cm beam and then the 1cm beam. On training day 2, each mouse was trained to traverse the horizontal 1cm beam and then the 1cm beam inclined at an angle of 10° from ground. Tree training sessions par beam and the mice rest for 10 min in their home cages between training sessions on the two beams. On the day test, time taken to traverse the 1cm beam inclined at an angle of 10° from ground and the number of paw faults or slips are recorded. Analysis of each measure was based on the mean scores of the two best trials.

*Grid handling.* Mice were placed on a metal grid (spacing 1 cm2) and allowed to grip the grid with four paws. The grid was inverted at an angle of 180°, 20 cm above the ground and the time for the mouse to fall onto soft bedding was measured in seconds.

*Morris water maze.* Spatial learning and memory were performed as described previously ^10^ except for the reversal acquisition training where the platform was moved to the opposite quadrant used previously for the spatial learning test.

*Fear conditioning.* On day1, mice were trained in a standard fear conditioning apparatus. The training consisted of a single trial and was performed in a constantly illuminated Plexiglas cage (transparent walls and metal grid floor). Fear conditioning was performed by placing the mice in context A for 180s acclimation period followed by three conditional-unconditional stimulus (CS-US) separated by 120s interval. The CS-US consisted of 3 successive tones (CS, 20 s, 65 dB) and a footshock (US, 0.4 mA, 1 s, constant current) delivered through a stainless-steel grid floor. The fear conditioning chamber was thoroughly cleaned with 70% ethanol before each mouse was placed in the box. Memory for the context and the tone were evaluated on day 2 (recent memory) or 8 (remote memory) following conditioning. For the contextual memory test, mice were placed into the conditioning chamber and allowed to explore for 360 sec. For the tone memory test, the same mice were placed in a novel cage (cage with colored walls and flat plastic floor) 3 hr after the contextual memory test, allowed to acclimate to the chamber for 180sec and then presented with tone (180 sec, 65 dB). Freezing, an index of fear defined as the lack of movement except for heart-beat and respiration, was recorded during the 180sec acclimation period and context or tone presentation ^11^.

# Supplementary references

1. Madisen, L. *et al.* A robust and high-throughput Cre reporting and characterization system for the whole mouse brain. *Nat. Neurosci.* **13**, 133–140 (2010).

2. Yamben, I. F. *et al.* Scrib is required for epithelial cell identity and prevents epithelial to mesenchymal transition in the mouse. *Dev. Biol.* **384**, 41–52 (2013).

3. Hilal, M. L. *et al.* Activity-Dependent Neuroplasticity Induced by an Enriched Environment Reverses Cognitive Deficits in Scribble Deficient Mouse. *Cereb. Cortex* **27**, 5635–5651 (2017).

4. Ezan, J. *et al.* Primary cilium migration depends on G-protein signalling control of subapical cytoskeleton. *Nature Cell Biology* **15**, 1107–1115 (2013).

5. Hébert, J. M. & McConnell, S. K. Targeting of cre to the Foxg1 (BF-1) locus mediates loxP recombination in the telencephalon and other developing head structures. *Dev. Biol.* **222**, 296–306 (2000).

6. Gorski, J. A. *et al.* Cortical excitatory neurons and glia, but not GABAergic neurons, are produced in the Emx1-expressing lineage. *J. Neurosci.* **22**, 6309–6314 (2002).

7. Zhou, L. *et al.* Early Forebrain Wiring: Genetic Dissection Using Conditional Celsr3 Mutant Mice. *Science* **320**, 946–949 (2008).

8. Montcouquiol, M. *et al.* Asymmetric localization of Vangl2 and Fz3 indicate novel mechanisms for planar cell polarity in mammals. *J. Neurosci.* **26**, 5265–5275 (2006).

9. Calderon de Anda, F. *et al.* Autism spectrum disorder susceptibility gene TAOK2 affects basal dendrite formation in the neocortex. *Nature Neuroscience* **15**, 1022–1031 (2012).

10. Moreau, M. M. *et al.* The planar polarity protein Scribble1 is essential for neuronal plasticity and brain function. *J. Neurosci.* **30**, 9738–9752 (2010).

11. Rivero, O. *et al.* Cadherin-13, a risk gene for ADHD and comorbid disorders, impacts GABAergic function in hippocampus and cognition. *Transl Psychiatry* **5**, e655 (2015).

**Supplementary Figure legends**

**Supplementary Figure S1. Circletail mutants exhibit severe neural tube defects / craniorachischisis, impeding brain development analysis.** **A**, E16.5 control littermate and **B**, Circletail Crc/Crc heterozygous embryos in lateral view. Coronal sections of **C,** control and **D,** Crc/Crc mutant, performed at the level of the dashed lines in A, B, and stained with hematoxylin. Scale Bar: 100µm.

**Supplementary Fig S2. Microcephaly and severe cortical layering defects in *FoxG1-Scrib***^-/-^ **cKO brains. A**, Dorsal views of P0 *FoxG1-Scrib*^-/-^ cKO brains. Dorsal cortical surface areas are outlined with a yellow dashed line. Statistical analysis via a two-tailed t test (*P*<0.05*) using between 5 and 8 brains per genotype from at least 3 independent experiments. Error bars indicate the SD. Scale bar: 1 mm. **B**, Schematic view of a P0 brain sectioned coronally at the rostral (R.) or caudal (C.) level. **C-D**, Representative hematoxylin staining of coronal sections from newborn *FoxG1-Scrib*^-/-^ cKO motor cortex at the caudal (C) and rostral (D) levels and their respective controls. A marked reduction of the motor cortex thickness (M) in cKOs extends to the cingulate (Cg) and somatosensory (S1-S2) cortex at both rostral (C) and caudal (D) levels. Cortical plate thickness was measured radially from the top of the upper layer (UL) to the bottom of the lower layer (LL) of the cortex. IZ: Intermediate Zone, SVZ: Sub-Ventricular Zone, VZ: Ventricular Zone. Statistical analysis via a two-tailed t test (*P***<0.0001*) using between 6 to 8 measurements per genotype from at least 3 independent experiments. Error bars indicate the SEM. Scale bar: 0.2 mm. **E-G**, Representative Immunofluorescence staining of CuxI (E), Satb2 (F) and Ctip2 (G) on coronal sections from newborn *FoxG1-Scrib*^-/-^ cKO brains in caudal motor cortex. Quantification of CuxI-, Satb2- and Ctip2-positive neurons is shown as a percentage (see methods). Severe reduction of CuxI (ctrl, 29.9% ± 3.4; mutant, 9.75% ± 2.3; *p* = 0.003), Satb2 (ctrl, 40.5% ± 2.2; mutant, 17.9% ± 2.4; *p* = 0.001) and Ctip2 (ctrl, 11.4% ± 0.8; mutant, 6.1% ± 0.9; *p* = 0.006) percentages were observed in *FoxG1-Scrib*^-/-^ cKO caudal motor cortices. Statistical analysis via a two-tailed t test (*P*<0.05, P**<0.01)* using between 3 to 4 measurements per genotype from at least 3 independent experiments. Error bars indicate the SD. **H**, Schematic representation of cortical layering in caudal motor cortex of *FoxG1-Scrib*^-/-^ cKO and its control. Both early-born (brown) and late-born neurons (blue) are massively decreased in proportion suggesting cell fate defects. See also Fig3.

**Supplementary Fig S3. Complete corpus callosum agenesis in *FoxG1-Scrib***^-/-^ ***cKO* mutants. A-D**, Representative hematoxylin staining of coronal sections from newborn *FoxG1-Scrib*^-/-^ cKO brains (B and D) and their respective controls (A and C) at the caudal (A-B) or rostral (C-D) levels. **A’-D’**, Higher magnification for selected insets (boxed areas) from (A-D) illustrating high penetrance of CC agenesis (ACC) both at the caudal and rostral level. At P0, 100% of *FoxG1-Scrib*^-/-^ (n=12) cKO brains displayed ACC. Instead of crossing the midline, CC axons formed whorls (Probst bundles, PB) on either side of the midline that are indicated with an asterisks in B’ and D’. **A’’-D’’**, DiI crystals placed in the dorsomedial cortex trace CC axons in *FoxG1-Scrib*^-/-^ cKO brains (B” and D”) and their respective controls (A” and C”) at the caudal (A”-B”) or rostral (C”-D”) levels at P0. In *FoxG1-Scrib*^-/-^ cKO brains, misrouted callosal axons form dense PB (asterisks in B”, D”) lateral to the midline. Abbreviations: Cortex (Cx), Hippocampus (Hp), Corpus Callosum (CC). Midline is indicated as a white dashed line. Scale bars: 1 mm in (A-D), 0.1 mm in (A’-D’).

**Supplementary Fig S4. Corpus callosum agenesis in *Scrib*** ***cKO* mutants is associated with impaired midline glia structure positioning. A-B’**, Representative immunofluorescence staining of L1-CAM (red) and GFAP (green) as a merged image (A-B) or GFAP only (A’-B’, gray) on coronal sections from newborn *Emx1-Scrib*^-/-^ (B and B’, orange) cKO brains together with their respective controls at the caudal level. In *Scrib* cKO brains, agenesis of the corpus callosum is confirmed by the failure of L1-CAM-positive axonal fibers to cross the midline. GFAP-positive midline glial structures such as the MZ are missing in these mutants, resulting in improper hemisphere fusion and failure for the IG to form and localize appropriately. **C-D**, Representative immunofluorescence staining of Scrib (green) and GFAP (red) as a merged image (C-D), GFAP only (C’-D’, gray), DAPI only (C’’-D’’, gray) or Scrib only (C’’’-D’’’, gray) on coronal sections from P2 *Emx1-Scrib*^-/-^ (B and B’, orange) cKO brains. Scrib is enriched in the GFAP-positive cells in the control brains while it is virtually absent from the dorsal structures of the mutant brain (except in the choroid plexus, see *), hence absent from the scattered GFAP-positive cells. **E-F’**, Representative immunofluorescence staining of L1-CAM (red) and GFAP (green) as a merged image (E-F) or GFAP only (E’-F’, gray) on coronal sections from newborn *FoxG1-Scrib*^-/-^ (F and F’, red) cKO brains together with their respective controls at the caudal level. There is a similar phenotype than in *Emx1-Scrib*^-/-^ cKO brains. Scale bars: 0.1 mm in (A-F).

**Supplementary Fig S5.** **ACC is accompanied by hippocampal commissure agenesis in *Scrib***^-/-^ **cKO mutants. A-B,** Representative hematoxylin staining of para-sagittal sections from newborn *FoxG1-Scrib*^-/-^ KO brains (B) and its control (A). Commissural plates are magnified in each inset. The AC (indicated by arrowheads) is still present in brains from cKO mutants. **C**, Marked reduction of CC length (in mm) along the rostro-caudal axis in *FoxG1-Scrib*^-/-^ cKO brains (red, n=8) as compared with their control littermates (white, n=6). Statistical analysis via a two-tailed t test (*P***<0.0001*) using between 5 and 8 brains per genotype from at least 3 independent experiments. **D-G**, Representative hematoxylin staining of serial horizontal sections from newborn *FoxG1-Scrib*^-/-^ (E,G) and their respective controls (D,F) at the dorsal (D-E) and ventral level (F-G). **D’-G’**, Higher magnification for selected insets (boxed areas) from (D-G). Sections through the *FoxG1-Scrib*^-/-^ cKO brains reveal the complete absence of the CC and DHC. Misrouted callosal axons form dense PB (asterisks) lateral to the midline in dorsal sections and failed to reach the contolateral hemisphere ventrally (E’, asterisk). Despite an apparent thinning, the VHC is still observed in ventral sections (G’).These defects were penetrant in all *Scrib*^-/-^ cKO mutants observed (n=3 for each genotype). Scale bars: 1 mm in (A-B, D-G), 0.2 mm in (D’-G’).

**Supplementary Fig S6.** **Full scans of western blots shown in Figure 2C**
